# Is Thrombectomy Effective for Large Vessel Occlusion Stroke Patients with Mild Symptoms? Meta-Analysis and Trial Sequential Analysis

**DOI:** 10.3390/life14101249

**Published:** 2024-10-01

**Authors:** Kuan-Chih Chen, Te-Wei Li, Ji-Kuan Huang, Cheng-Chieh Huang, Siang-Yan Zhang, Chih-Hung Chen, Zong-Syuan Lin, Po-Huang Chen, Hong-Jie Jhou

**Affiliations:** 1 Department of Emergency and Critical Care Medicine, Changhua Christian Hospital, Changhua 50006, Taiwan; 182917@cch.org.tw (K.-C.C.); 183382@cch.org.tw (T.-W.L.); 184431@cch.org.tw (J.-K.H.); 169305@cch.org.tw (C.-C.H.); 182904@cch.org.tw (Z.-S.L.); 2 School of Medicine, Kaohsiung Medical University, Kaohsiung 80756, Taiwan; 185008@cch.org.tw (S.-Y.Z.); 184477@cch.org.tw (C.-H.C.); 3 Division of General Practice, Department of Medical Education, Changhua Christian Hospital, Changhua 50006, Taiwan; 4 Division of Hematology and Oncology, Department of Internal Medicine, Tri-Service General Hospital, National Defense Medical Center, Taipei 11490, Taiwan; 5 Department of Neurology, Changhua Christian Hospital, 135 Nanhsiao Street, Changhua 50006, Taiwan

**Keywords:** acute ischemic stroke, endovascular thrombectomy, large vessel occlusion, low NIHSS score, meta-analysis

## Abstract

Background: Endovascular treatment (EVT) is an established method for managing large vessel occlusion (LVO), but its efficacy in patients with mild stroke (National Institutes of Health Stroke Scale [NIHSS] score < 6) remains debated. Given the clinical problem of early neurological deterioration in approximately 10% of mild stroke patients, understanding the role of EVT in managing these patients is crucial. Our objective was to perform a meta-analysis with trial sequential analysis (TSA) focusing on mild stroke patients with LVO to determine whether EVT offers better outcomes than best medical therapy alone. Methods: A comprehensive search of PubMed, Cochrane, and Embase databases up to 12 December 2023 identified 14 retrospective and prospective cohort studies, including a total of 4436 patients with NIHSS scores less than 6 and presenting with LVO. Studies were categorized into crossover and non-crossover groups to prevent overestimation of the treatment effect. In the crossover group, patients initially treated with BMT were moved to EVT upon clinical deterioration. In the non-crossover group, patients remained in their initially assigned treatment. Meta-analysis and data extraction followed the Preferred Reporting Items for Systematic Reviews and Meta-Analyses guidelines. The primary outcome was achieving an excellent functional outcome, defined as a modified Rankin scale (mRS) score of 0–1 at 3 months. Secondary outcomes included good (mRS 0–2) and favorable (mRS 0–3) functional outcomes. Safety outcomes were symptomatic intracerebral hemorrhage (sICH) and mortality at 3 months. Results: In the crossover group, EVT did not significantly improve excellent functional outcomes, and TSA results were inconclusive. Conversely, in the non-crossover group, EVT significantly improved the excellent functional outcome rates at 3 months (65.0% vs. 53.7%; OR 1.62; 95% CI 1.13 to 2.32), supported by TSA. EVT increased the risk of sICH in both crossover and non-crossover groups, while mortality rates did not significantly differ between EVT and BMT groups. Conclusions: Our research indicates that thrombectomy may not significantly help mild stroke patients in recovering functional status and could increase the risk of sICH. The disparity in results between crossover and non-crossover studies highlights the critical need for the prompt identification of patients at risk of early neurological deterioration to minimize negative outcomes. Additional randomized controlled trials are essential to optimize the application of EVT in this patient population.

## 1. Introduction

Endovascular treatment (EVT) has been shown to be effective for managing large vessel occlusion (LVO) across both the anterior and posterior circulations and is therefore considered the gold standard for managing this condition [1,2]. Landmark trials confirming the effectiveness of thrombectomy followed several unsuccessful studies, and consequently, rigorous criteria for selecting patients have been adopted; notably, most studies [3,4,5,6,7,8] have excluded patients with mild symptoms, except the MR CLEAN trial, which included 10 patients with a National Institutes of Health Stroke Scale (NIHSS) score of ≥2, and the EXTEND-IA trial, which enrolled 4 patients without NIHSS score restrictions. Given that approximately 10% of patients diagnosed with mild stroke may experience concurrent LVO, the suitability of EVT for these patients requires further investigation [9]. A cohort study pooling data from the EXTEND-IA study and various EVT centers revealed that patients with a target mismatch profile may have an increased likelihood of functional independence [10]. Among patients in whom stroke was identified within a late window, the MR CLEAN-LATE study [11], involving 142 participants with low NIHSS scores (2–6), showed that although thrombectomy improved outcomes overall, there were no significant differences in outcomes among the subgroups with low NIHSS scores. In addition to thrombectomy, thrombolysis may be an alternative option for these patients [12]. However, approximately 10–20% of patients without reperfusion therapy may experience early neurological deterioration [13], and resorting to rescue EVT may result in less favorable outcomes [14]. Consequently, clinicians face a dilemma in providing immediate EVT in this scenario.

A meta-analysis [15] indicated that thrombectomy in patients with mild stroke following LVO did not improve functional outcomes but rather increased the risk of symptomatic intracranial hemorrhage, showing that primary EVT may not be beneficial for this specific stroke subgroup. However, several recent cohort studies have presented varying results on this subject. Yedavalli et al. [16] showed that EVT improved clinical outcomes, while Cappellari et al. [17] found that combining EVT with intravenous thrombolysis did not yield better outcomes compared to thrombolysis alone. Given these discrepancies, we propose a meta-analysis to offer updated insights on the use of EVT for these patients. The objective of this study was to evaluate patients with NIHSS scores less than 6 following LVO to determine whether primary thrombectomy yielded better clinical outcomes than medical management alone. Additionally, we planned to perform trial sequential analysis (TSA) to obtain more precise outcomes and mitigate the risk of overestimation.

## 2. Materials and Methods

This research was duly registered with the International Prospective Register of Systematic Reviews (PROSPERO) (available at CRD42024509166) and adhered to the reporting standards outlined in the Preferred Reporting Items for Systematic Reviews and Meta-Analyses (PRISMA) [18] (Online Supplemental Table S1) and the Meta-analysis of Observational Studies in Epidemiology (MOOSE) [19] (Online Supplemental Table S2). These standards are pivotal for the methodical execution of meta-analyses and reviews pertinent to observational studies. To facilitate reproducibility and foster transparency in the scientific community, we have provided all essential data within this publication, allowing other researchers to validate and replicate our findings.

### 2.1. Search Strategy

Our search strategy involved a thorough review of literature across the PubMed, Embase, and Cochrane Library databases, covering all records up to 12 December 2023, without language restrictions. We employed a strategic combination of keywords, specifically “stroke”, “NIHSS < 6”, and “endovascular treatment”, alongside other potentially pertinent keywords (Online Supplemental Table S3). To ensure thoroughness and to capture any studies that might not have been indexed in the aforementioned databases, we also manually searched the references cited within the selected articles and other relevant publications. For studies deemed eligible for inclusion in our review but lacking original data, the corresponding authors were contacted to obtain the missing information.

### 2.2. Study Selection

This study focused on the outcomes of stroke patients with LVO who presented with an NIHSS score less than six, and articles were selected if they compared those who received EVT with those who received best medical therapy (BMT) alone. Studies were excluded if the patients had an NIHSS score of six, if they exclusively investigated distal, medium vessel occlusions, if they lacked a control group, or if they involved indirect comparisons due to population differences. Furthermore, conference abstracts, posters, and studies with potential patient data overlap were not included, ensuring that the most recent publications were prioritized to ensure data accuracy and prevent duplication of patient counts.

### 2.3. Outcome Measurements

Our primary outcome focused on achieving an excellent functional patient outcome, defined by a modified Rankin scale (mRS) score of 0–1 at 3 months. The secondary outcomes included good (mRS score 0–2) and favorable (mRS score 0–3) functional patient outcomes at the same intervals. Safety outcomes were assessed according to the incidence of symptomatic intracerebral hemorrhage (sICH) and mortality at 3 months.

### 2.4. Data Extraction

Two investigators, KCC and TWL, conducted an initial screening of all abstracts of the selected articles to exclude irrelevant articles. The full texts of the relevant articles were subsequently obtained, and key information, including the first author’s name, study design, sample size, participant characteristics, presence of rescue EVT, and thrombolytic agent administration rate, was extracted. The meta-analysis utilized an intention-to-treat design to obtain a comprehensive understanding of treatment impact, despite the potential for larger effect sizes with a per-protocol design. Patients were categorized into two groups based on how rescue EVT was employed in each study: for studies that placed patients who were initially treated with BMT but who subsequently received rescue EVT upon deterioration into the EVT group, the patients were classified as the crossover group; for studies that placed patients who received rescue EVT into the BMT group, the patients were classified as the non-crossover group.

### 2.5. Risk of Bias Assessment

Two unbiased authors (ZSL and CHC) independently evaluated the risk of bias of each study using the Newcastle–Ottawa Scale [20], categorizing them as high (scores < 4), intermediate (scores 4–6), or low risk (scores ≥ 7–9).

### 2.6. Statistical Analysis

For binary outcomes, odds ratios (ORs) and their 95% confidence intervals (CIs) were considered the primary summary measures. Given the potential variability of true effect sizes across studies, random-effects models were adopted for pooled estimates, applying the DerSimonian and Laird method [21,22]. A fixed-effects model was considered if the between-study variance was zero. Study heterogeneity was assessed using the Cochran Q test [23] (expressed as the corresponding Cochran Q *p* value) and I^2^ statistics [24], categorizing heterogeneity into moderate (I^2^ ≥ 30%), substantial (I^2^ ≥ 50%), or considerable (I^2^ ≥ 75%).

Regarding the effects of rescue therapy, we separately estimated the outcomes for the crossover group and the non-crossover group. For studies with a high or intermediate risk of bias (Newcastle–Ottawa scale scores ≤ 6), a sensitivity analysis was conducted. Publication bias was demonstrated visually using funnel plots and quantitatively using Egger’s test via funnel plot asymmetry [25]. All the statistical tests were two-tailed with a significance threshold of *p* < 0.05 and were conducted using the “metafor” [26] and “meta” packages in R software version 4.0.1 [27].

### 2.7. Quality Assessment

The certainty of evidence for all outcomes in our study was assessed through the Grading of Recommendations Assessment, Development, and Evaluation (GRADE) [28] methodology using GRADEpro software (version 20, McMaster University, Hamilton, ON, Canada, 2014).

### 2.8. Trial Sequential Analysis

Meta-analyses often suffer from limited data and repeated testing, leading to a greater possibility of type I and type II errors. Notably, significant outcomes from a meta-analysis do not inherently guarantee sufficient statistical power to represent the true effect sizes accurately. This limitation necessitates the application of TSA, a method designed to mitigate the increased risk of random errors in meta-analyses. TSA rigorously evaluates the reliability and conclusiveness of accumulated evidence [29]. The methodology hinges on the trajectory of the cumulative z-curve; when this curve intersects with the trial sequential monitoring boundary or the requisite information boundary or ventures into the futility zone, the gathered evidence is implied to be substantial enough to corroborate the hypothesized intervention effect. Conversely, if the z-curve neither intersects with any boundary nor reaches the requisite information size, the evidence is considered insufficient, warranting further investigation [30].

To tailor TSA for meta-analysis with greater precision, the concept of diversity-adjusted required information size is introduced. This advanced approach integrates effect size, relative risk reduction, and the calculation of the respective chances of type I and type II errors. In our implementation of TSA, we determined the required information size through the use of a two-sided type I error (α) of 0.05 and a type II error (β) of 0.20, corresponding to 80% power, following the O’Brien–Fleming alpha-spending function approach. The relative risk reduction derived from the event rates in both the experimental and control groups was calculated from the average event proportions. Heterogeneity correction, tailored to the specific variance model, was applied. This analysis was conducted using the fixed-effect model in TSA software (version 0.9.5.1 Beta; Copenhagen Trial Unit, Copenhagen, Denmark).

## 3. Results

### 3.1. Search Detail

**Figure 1:**
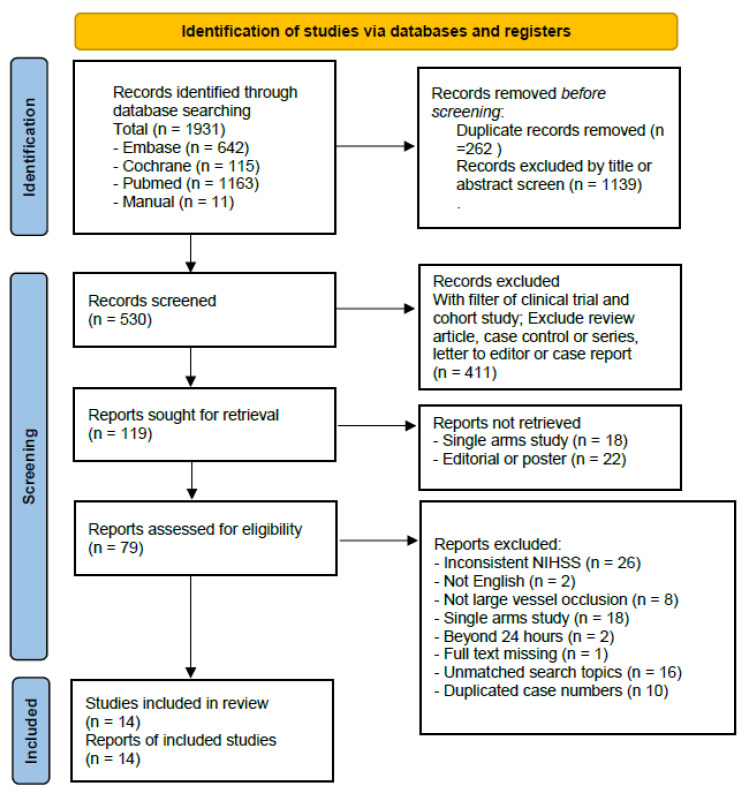
PRISMA 2020 flow diagram for new systematic reviews, which included searches of databases, registers, and other sources.

A total of 1931 references were identified via a search of the three electronic databases, which was narrowed to 119 potentially suitable articles following intensive screening and elimination of duplicates or irrelevant material. Subsequent in-depth evaluation led to further study elimination until 14 studies [10,16,17,31,32,33,34,35,36,37,38,39,40,41] remained for inclusion into the quantitative synthesis (Figure 1). These studies collectively included 4436 patients with mild stroke induced by LVO, regardless of whether they received EVT. The characteristics of these studies, including 2 prospective cohort studies and 12 retrospective cohort studies, are displayed in Table 1. Among all patients with mild stroke resulting from LVO, 1366 underwent EVT, while 3070 did not. The average age of these patients was 68 years (study-level range, 59.3–71.0). The initial mean NIHSS score was 3.3 (study-level range, 2–5). Not all studies specify EVT types, but those that do mention thrombectomy, angioplasty, stenting, intra-arterial thrombolysis, or a combined approach [10,33,34,36,38,40]. Some studies indicate that within the EVT group, patients tend to have fewer vascular risk factors, more proximal occlusions, and higher NIHSS scores [10,31,34,39]. Additionally, among the studies, 12 provided data on the number of patients receiving rescue EVT, totaling a proportion of 6.9% (179 out of 2606).

### 3.2. Primary Outcomes

#### Excellent Functional Outcomes

**Figure 2:**
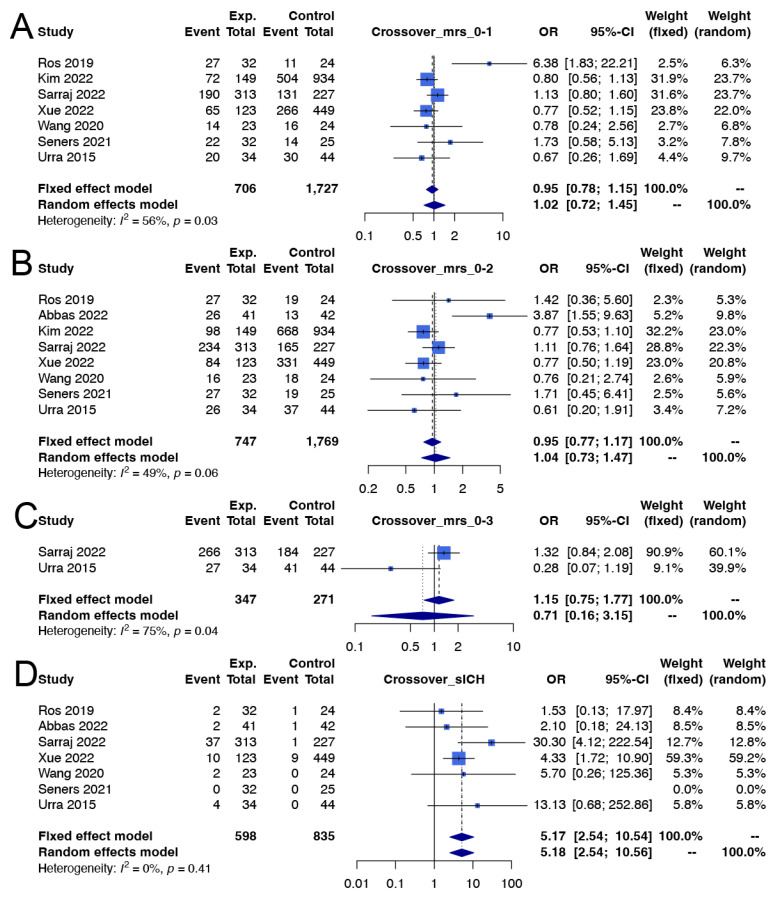

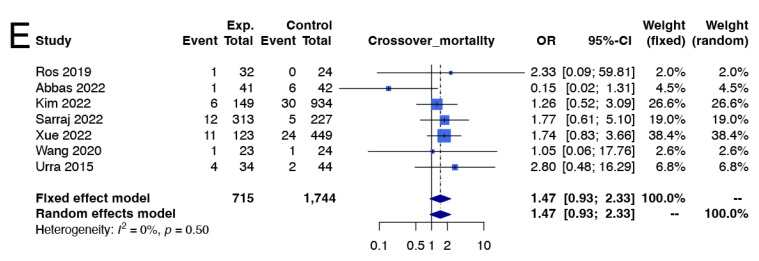
Forest plots showing the effectiveness of thrombectomy for mild stroke patients in studies with a crossover design. (**A**) Excellent functional outcome, (**B**) good functional outcome, (**C**) favorable functional outcome, (**D**) symptomatic intracranial hemorrhage, (**E**) mortality. [10,31,33,35,36,38,39,40].

**Figure 3:**
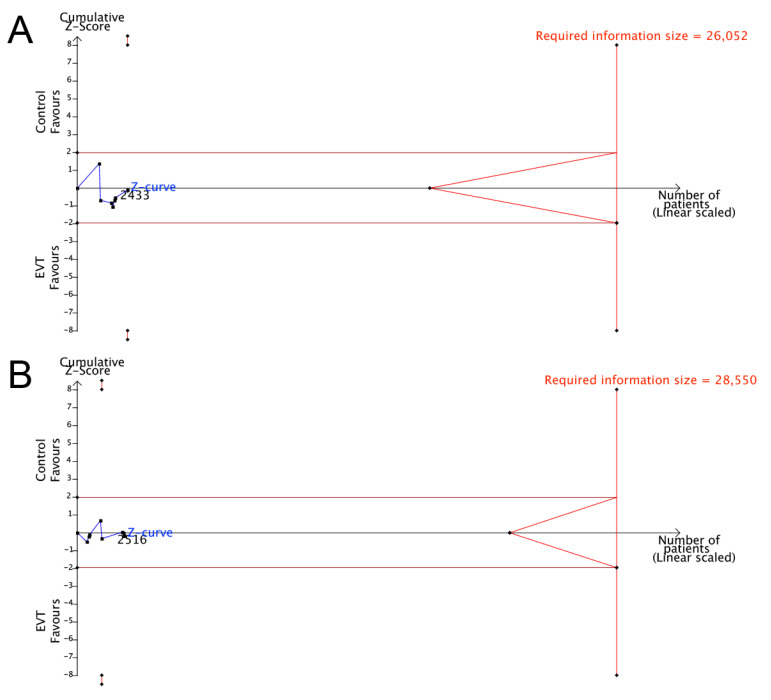

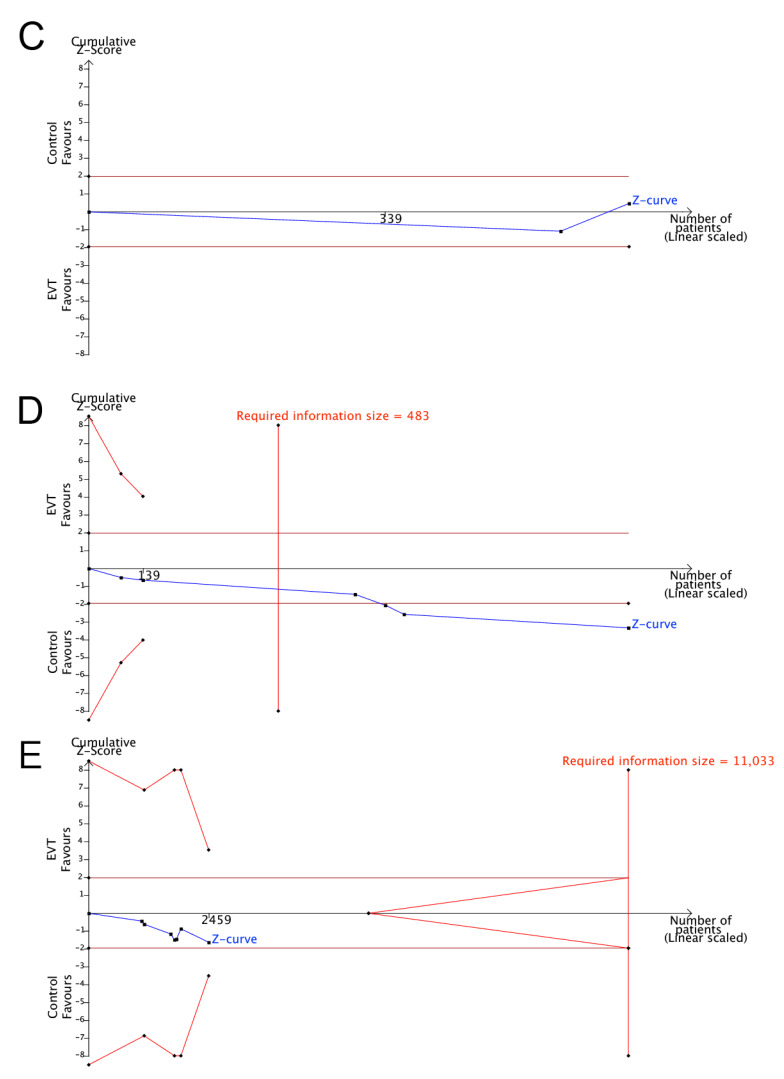
Trial sequential analysis of outcomes in crossover-design studies. (**A**) Excellent functional outcome, (**B**) good functional outcome, (**C**) favorable functional outcome, (**D**) symptomatic intracranial hemorrhage, (**E**) mortality. The *X*-axis represents the total number of patients included in the analysis, and the *Y*-axis indicates the cumulative Z score. Horizontal dark red lines mark the conventional boundaries used to determine statistical significance. The sloping red lines at the top and bottom left-hand corners, also known as trial sequential boundaries, signify the thresholds required for statistical significance within the context of TSA. Red diagonal lines within the horizontal brown lines delineate the futility boundaries, suggesting areas where continuing the trial is unlikely to yield significant results. The full vertical red line indicates the required information size, the total amount of data needed to reach a conclusive result. The solid blue line represents the cumulative Z-curve, showing the progression of the evidence of the statistical test over the course of the study.

Data from ten cohort studies comprising 4353 patients were analyzed regarding excellent functional outcomes at 3 months. In the crossover group [10,31,33,35,36,39,40], the use of EVT did not significantly improve the excellent functional outcome rates with respect to control treatment (58.1% vs. 56.3%; OR 1.02; 95% CI 0.72 to 1.45; I^2^ = 56%; *p* value of the Cochran Q test for heterogeneity = 0.03; Figure 2A). According to TSA, in the evaluation for excellent functional outcomes, the cumulative z-curve for the crossover group did not cross the required information size or the trial sequential monitoring boundary, indicating that the results were inconclusive (Figure 3A).

**Figure 4:**
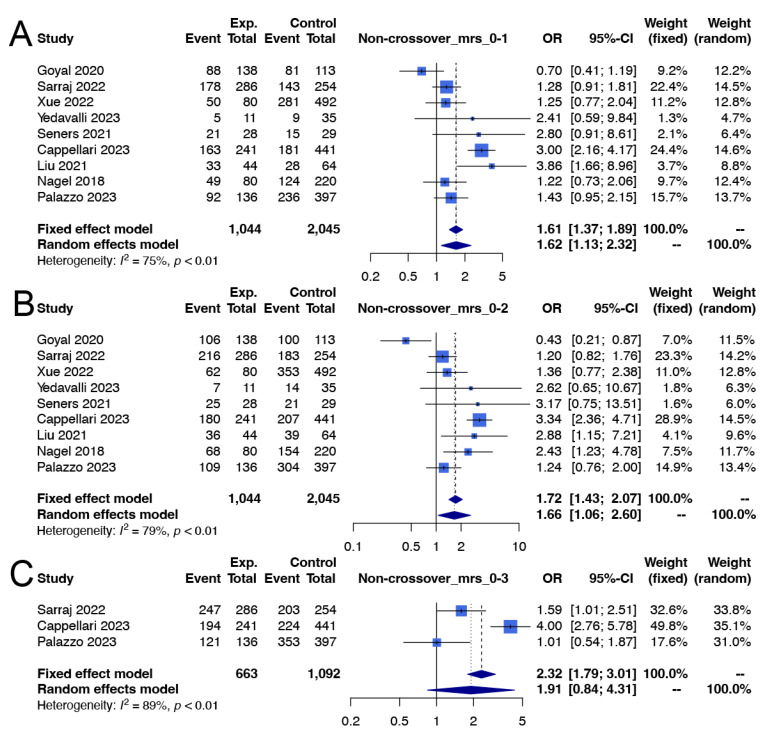

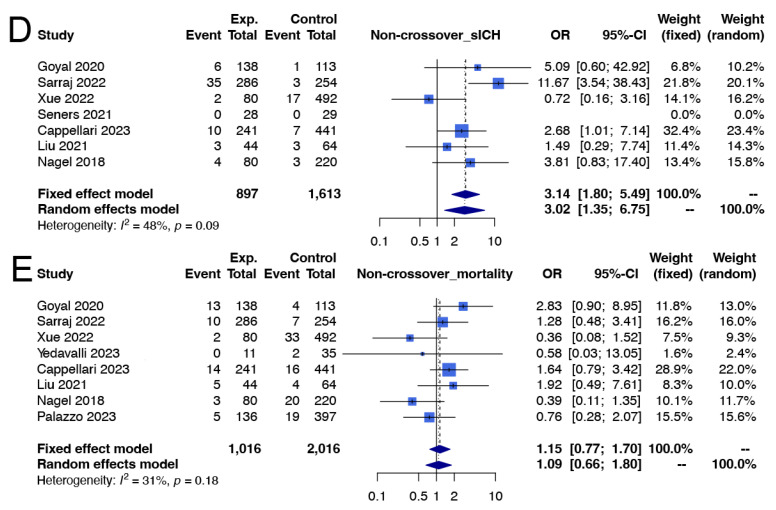
Forest plots showing the effectiveness of thrombectomy for mild stroke in patients from studies with a non-crossover design. (**A**) Excellent functional outcome, (**B**) good functional outcome, (**C**) favorable functional outcome, (**D**) symptomatic intracranial hemorrhage, (**E**) mortality. [10,16,17,32,34,36,37,40,41].

**Figure 5:**
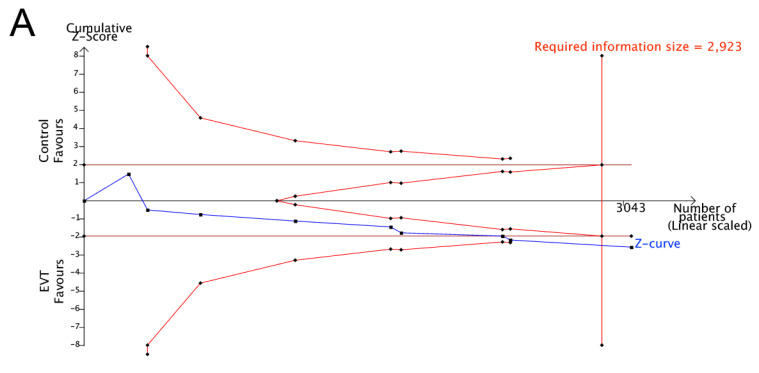

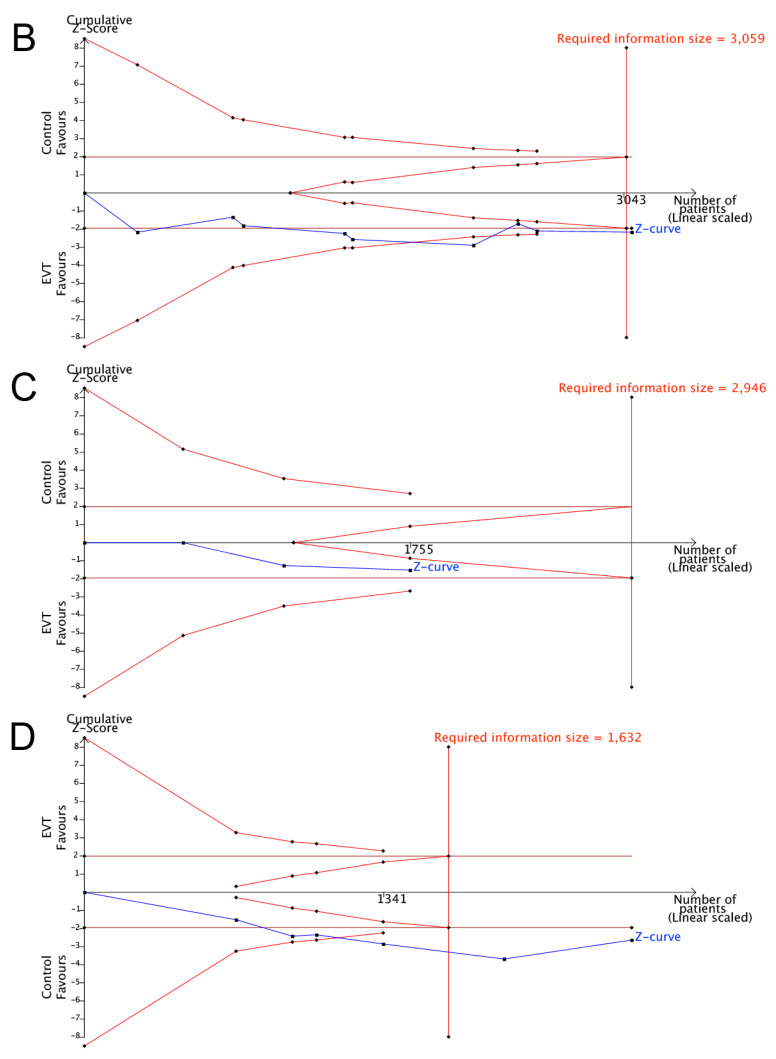

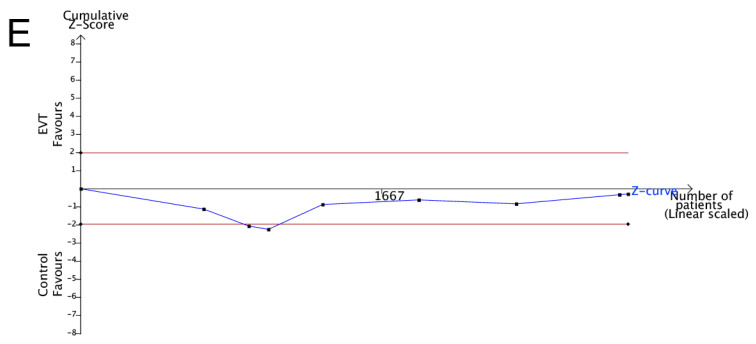
Trial sequential analysis of patient outcomes in studies with a non-crossover design. (**A**) Excellent functional outcome, (**B**) good functional outcome, (**C**) favorable functional outcome, (**D**) symptomatic intracranial hemorrhage, (**E**) mortality. The *X*-axis represents the total number of patients included in the analysis, and the *Y*-axis indicates the cumulative Z score. Horizontal dark red lines mark the conventional boundaries used to determine statistical significance. The sloping red lines at the top and bottom left-hand corners, also known as trial sequential boundaries, signify the thresholds required for statistical significance within the context of TSA. Red diagonal lines within the horizontal brown lines delineate the futility boundaries, suggesting areas where continuing the trial is unlikely to yield significant results. The full vertical red line indicates the required information size, the total amount of data needed to reach a conclusive result. The solid blue line represents the cumulative Z-curve, showing the progression of the evidence of the statistical test over the course of the study.

Conversely, in the non-crossover group [10,16,17,32,34,36,37,40,41], EVT recipients demonstrated a significantly better excellent functional outcome rate than nonrecipients did (65.0% vs. 53.7%; OR 1.62; 95% CI 1.13 to 2.32; I^2^ = 75%; *p* value of the Cochran Q test for heterogeneity < 0.01; Figure 4A). According to TSA, in the evaluation for excellent functional outcomes, the cumulative z-curve for the non-crossover group crossed the required information size and the conventional boundary, favoring the use of EVT. This indicates that the results were conclusive and provides solid statistical evidence to support our findings (Figure 5A).

### 3.3. Secondary Outcomes

#### Good Functional Outcome and Favorable Functional Outcome

In the crossover group, EVT did not significantly affect the good functional outcome [10,31,33,35,36,38,39,40] (72.0% vs. 71.8%; OR 1.04; 95% CI 0.73 to 1.47; I^2^ = 49%; *p* value of the Cochran Q test for heterogeneity = 0.06; Figure 2B) or favorable functional outcome rate [10,31] (84.4% vs. 83.0%; OR 0.71; 95% CI 0.16 to 3.15; I^2^ = 75%; *p* value of the Cochran Q test for heterogeneity = 0.04; Figure 2C). According to TSA, in the evaluation for both good and favorable functional outcomes, the cumulative z-curve for the crossover group did not cross the required information size or the trial sequential monitoring boundary, indicating that the results were inconclusive (Figure 3B,C).

Conversely, in the non-crossover group, patients who underwent EVT exhibited a significantly better good functional outcome rate [10,16,17,32,34,36,37,40,41] (77.5% vs. 67.2%; OR 1.66; 95% CI 1.06 to 2.60; I^2^ = 79%; *p* value of the Cochran Q test for heterogeneity < 0.01; Figure 4B). However, the rates of favorable functional outcomes [10,17,41] were not significantly different between treatments (84.8% vs. 71.4%; OR 1.91; 95% CI 0.84 to 4.31; I^2^ = 89%; Cochran Q *p* value for heterogeneity < 0.01; Figure 4C). According to TSA, in the evaluation for good functional outcomes, the cumulative z-curve for the non-crossover group crossed the required information size and the conventional boundary, favoring EVT. This finding suggests a statistically significant result and provides strong evidence to support our findings. However, when the same non-crossover group was evaluated for favorable functional outcomes, the cumulative z-curve did not cross the required information size or the trial sequential monitoring boundary, indicating that the results in this aspect were inconclusive (Figure 5B,C).

### 3.4. Safety Outcomes

#### sICH and Mortality at 3 Months

In both the crossover [10,31,33,35,36,38,40] and non-crossover groups [10,17,32,34,36,37,40], use of EVT was significantly associated with a greater risk of sICH in the crossover group (9.5% vs. 1.4%; OR 5.17; 95% CI 2.54 to 10.54; I^2^ = 0%; *p* value of the Cochran Q test for heterogeneity = 0.41; Figure 2D) and in the non-crossover group (6.7% vs. 2.1%; OR 3.02; 95% CI 1.35 to 6.75; I^2^ = 48%; *p* value of the Cochran Q test for heterogeneity = 0.09; Figure 4D). According to TSA, for both the crossover and non-crossover groups regarding the outcomes of sICH, the cumulative z-curves crossed the required information size and the conventional boundary, favoring EVT. This indicates that the results were conclusive and provides solid statistical evidence to support our findings (Figure 3D and Figure 5D).

Mortality rates at 3 months posttreatment, however, were not significantly different between patients who underwent EVT and those who did not for either the crossover [10,31,33,35,38,39,40] (5.0% vs. 3.9%; OR 1.47; 95% CI 0.93 to 2.33; I^2^ = 0%; *p* value of the Cochran Q test for heterogeneity = 0.50; Figure 2E) or non-crossover group [10,16,17,32,34,37,40,41] (5.1% vs. 5.2%; OR 1.09; 95% CI 0.66 to 1.80; I^2^ = 31%; *p* value of the Cochran Q test for heterogeneity = 0.18; Figure 4E). According to TSA, regarding mortality in both the crossover and non-crossover groups, the cumulative z-curve did not cross the required information size or the trial sequential monitoring boundary. This indicates that the results in this aspect were inconclusive (Figure 3E and Figure 5E).

### 3.5. Sensitivity Analysis, Risk of Bias Assessment, and Assessment of Study Quality

We excluded studies that had a high or intermediate risk for bias in the sensitivity analysis, which did not lead to any significant differences in the rates of excellent or good functional outcomes or sICH within the non-crossover group but rather reduced the heterogeneity [10,34,36,37,40,41] (Online Supplemental Figure S2). The other outcomes for both the non-crossover and crossover groups remained consistent with the original findings in the sensitivity analysis (Online Supplemental Figure S2). The assessment of the risk of bias of all included studies is detailed in Online Supplemental Table S4: 11 studies were categorized as high quality, and no publication bias was found for any of the outcomes, confirming the reliability of the findings. Online Supplemental Table S5 summarizes the GRADE assessments of all outcomes. In the crossover group, the certainty of evidence was high for sICH but low for all functional outcomes and mortality at 3 months. For the non-crossover group, the certainty of evidence was moderate for sICH, low for mortality at 3 months, and very low for all functional outcomes at 3 months.

## 4. Discussion

Our meta-analysis included 14 studies, including a total of 4436 patients and focusing on the effectiveness of EVT for patients with low NIHSS scores. Our findings in the non-crossover group showed significant improvements in outcomes, indicated by a greater proportion of patients who achieved an excellent or good functional outcome. In the crossover group, no significant improvement in functional outcomes was observed. These differences in our results might have been caused by the use of rescue EVT. Furthermore, thrombectomy can increase the risk of sICH without affecting mortality rates. However, after studies with high and intermediate risk of bias were removed, EVT did not improve the clinical outcome rates in either group. Furthermore, our study demonstrated that in all the control groups of patients with mild stroke induced by LVO, the rate of good functional outcomes reached 69.3%, which was higher than the 26.5% reported in previous LVO patient studies, suggesting limited potential for therapeutic intervention in such patients [1]. Concurrently, the incidence of sICH in all the EVT groups was approximately 7.8%, exceeding the 4.4% reported in prior studies. However, the mortality rate was lower, at 5%, than the previously reported 15.3% [1]. Therefore, the decision to perform EVT in mild stroke patients with LVO remains a topic worthy of further discussion, given the implications for patient outcomes and treatment efficacy.

In our study, we observed different outcomes between the non-crossover and crossover groups. Notably, in the non-crossover group, patients who received EVT exhibited significantly improved outcomes, while no such improvements were observed in the crossover group. We attribute this discrepancy primarily to the categorization of rescue EVT in each study. Patients undergoing rescue management often experience early neurological deterioration (END), and it is reasonable to anticipate poorer prognoses for these individuals [42]. This finding aligns with that from a study by Sarraj et al. [10], who noted that the prognosis following rescue EVT was inferior to that following primary EVT. Our study revealed a rescue EVT rate of only 6.9%, which is less than the previously reported rate of 10–20% for END [13,43]. This difference could have arisen from the nature of non-randomized controlled trials (RCTs) and the lack of standardized intervention methods in the referenced studies. Previous meta-analyses [15,44] have addressed rescue EVT, albeit subtly. We believe that the allocation of patients who underwent rescue EVT in the present study significantly influenced the baseline comparison between the EVT and BMT groups. Therefore, we further explored this issue and obtained consistent results in the non-crossover groups. This underscores the need for a more focused investigation into the matter rescue EVT, highlighting its impact on treatment outcomes and comparisons.

Given that patients who need rescue therapy often have worse outcomes, early identification of the need for primary EVT should be considered. The article by Sener et al. [45]. reported two independent predictors of END in mild stroke patients: occlusion location and thrombus length. Proximal occlusions were found to be associated with a greater risk of deterioration, a finding that was also supported by previous research [46]. Moreover, a longer thrombus length was correlated with an increased likelihood of END. Additionally, Sarraj et al. [10] noted in their cohort study that larger volumes of critically hypoperfused tissue, as visualized on imaging, were more likely to culminate in END. Hence, they proposed the importance of perfusion imaging in determining the necessity of EVT in patients with mild stroke caused by LVO. This perspective was echoed by Goyal et al. and Xue et al., who also called for the use of perfusion imaging in decision making [34,40]. The current guidelines for EVT beyond the time window reinforce this proposition and recommend relying on imaging to make informed decisions [47]. Furthermore, whether a mild stroke induced by LVO is considered disabling or nondisabling may also influence the risk of deterioration [48]. Consequently, experts from the European Society for Minimally Invasive Neurological Therapy suggest considering EVT in patients with disabling mild stroke resulting from LVO. However, current studies [41,49] have not identified consistent factors to guide this decision-making process. For patients with mild stroke caused by LVO, the decision of whether to administer EVT should be made at the individual level to maximize benefits, optimize the prognosis, and minimize the risk of adverse effects.

Our study findings should be interpreted with caution, acknowledging both the strengths and limitations inherent in our research. We updated our research to improve the statistical power and used TSA to balance type I and II errors, avoiding drawing premature conclusions. However, our study has several limitations. One primary concern is that differences in study designs, including time windows and occlusion sites, contributed to the moderate to high heterogeneity observed in the outcomes, and removing low-quality articles only slightly reduced this heterogeneity. Second, the primary endpoints varied across different articles, indicating that there remains no consensus on the primary goal for thrombectomy treatment in patients with mild stroke. Moreover, while EVT increased the risk of sICH, four studies employed the European Cooperative Acute Stroke Study (ECASS II) classification, three used the ECASS III criteria, and others applied parenchymal hemorrhage type 2 or any ICH combined with an increase in the NIHSS score of >4 points. These varying definitions for sICH hinder our ability to accurately detect a reasonable bleeding rate. Another notable limitation was that most studies did not clearly indicate the rationale for performing EVT or specify the timing of rescue EVT. Additionally, there was insufficient comparison to determine which type of EVT is superior. Finally, the observational nature of the included studies innately imbues them with less evidential weight than RCTs. Two forthcoming RCTs might elucidate these issues: the ENDOLOW trial (https://clinicaltrials.gov, accessed on 3 August 2024; unique identifiers: NCT04167527) and the MOSTE trial (https://clinicaltrials.gov, accessed on 3 August 2024; unique identifiers: NCT03796468). These trials are expected to provide results regarding thrombectomy for patients with mild stroke resulting from LVO, thereby enriching the clinical evidence for future treatment guidance.

## 5. Conclusions

Our study suggests that thrombectomy may not significantly aid mild stroke patients in regaining functional status and could increase the risk of sICH. Differences in outcomes between crossover and non-crossover studies confirm the importance of promptly identifying patients at risk of END to reduce poor prognosis. Further RCTs are required to refine the conditions under which EVT should be used in mild stroke patients to determine the conditions under which EVT may be beneficial for mild stroke patients.

## Figures and Tables

**Table 1:** Characteristics of included studies.

Author, Year	Study Design	Country	No. of Patients	Age (Year)	Female (%)	NIHSS	HTN (%)	DM (%)	AF (%)	Previous Stroke (%)	tPA (%)	Time Window	Occlusion Site	Successful Recanalization (%)	Rescue EVT (n)	Overall RoB
Urra [31], 2015	Prospective cohort study	Spain	EVT: 34 BMT: 44	64.0 ± 14.8 71.0 ± 12.5	29.4 40.9	4 (3–5) 3 (3–5)	61.8 65.9	17.6 13.6	26.5 31.8	14.7 4.5	47.1 65.9	0–6 h	ICA, M1 M2, Posterior Circulation	91.2 N/A	16 N/A	8
Nagel [32], 2018	Retrospective cohort study	USA	EVT: 80 BMT: 220	65.3 ± 13.5 69.5 ± 14.1	45.0 46.8	4 (0–5) 3 (0–5)	70.0 72.3	16.3 16.4	43.8 28.2	N/A N/A	47.5 51.8	N/A	ICA, M1 M2, ACA BA	87.5 92.0 *	N/A 25	6
Ros [33], 2019	Retrospective cohort study	Europe	EVT: 32 BMT: 24	70 (23–92) 68 (40–95)	55.0 37.0	4 (2–4) 4 (3.5–5)	76.0 71.0	3.0 25.0	34.0 17.0	N/A N/A	59.0 100.0	0–24 h	ICA, M1, M2	100.0 N/A	1 N/A	9
Goyal [34], 2020	Retrospective cohort study	Multi-national	EVT: 138 BMT: 113	65.2 (16.6) 64.8 (12.8)	47.1 45.1	4 (3–5) 3 (2–4)	74.6 70.7	28.9 25.9	29.4 21.2	N/A N/A	54.0 41.5	0–24 h	ICA, M1, M2	84.5 N/A	N/A N/A	8
Wang [35], 2020	Retrospective cohort study	China	EVT: 23 BMT: 24	59.3 ± 8.3 64.4 ± 10.4	26.1 29.2	3 (2–5) 3 (2.25–4)	74.0 70.8	17.4 25.0	8.7 8.3	17.4 37.5	69.6 100.0	N/A	ICA, M1 M2, VBA	95.7 N/A	4 N/A	8
Seners [36], 2021	Retrospective cohort study	France	EVT: 28 BMT: 29	67 (56–75) 71 (62–83)	36.0 38.0	4 (2–5) 4 (2–5)	55.0 54.0	7.0 10.0	14.0 17.0	N/A N/A	100.0 100.0	N/A	BA	60.7 50.0 *	N/A 4	7
Liu [37], 2021	Prospective cohort study	China	EVT: 44 BMT: 64	66.51 ± 9.26 67.02 ± 13.18	37.2 33.9	5 (4–5) 5 (3–5)	62.8 72.6	18.6 22.6	N/A N/A	16.3 12.9	44.2 41.9	0–24 h	ICA, M1, M2, BA, P1	97.7 N/A	N/A 2	8
Abbas [38], 2022	Retrospective cohort study	USA	EVT: 41 BMT: 42	66.12 ± 15.37 59.6 ± 16.19	39.0 47.6	N/A N/A	65.9 59.5	31.7 16.7	19.5 21.4	17.1 14.3	34.1 47.6	N/A	ICA, M1, A1	N/A N/A	3 N/A	7
Kim [39], 2022	Retrospective cohort study	South Korea	EVT: 149 BMT: 934	64.8 ± 12.6 67.6 ± 13.2	36.2 38.5	3 (1–4) 2 (1–4)	51.7 63.1	30.9 28.8	33.6 27.2	N/A N/A	1.3 12.3	0–24 h	ICA, M1, M2	N/A N/A	23 N/A	7
Sarraj [10], 2022	Retrospective cohort study	Multi-national	EVT: 286 BMT: 227 Rescue: 27	71 (59–80) 69 (58–76) 63 (53–75)	38.6 42.5 34.6	4 (2–5) 3 (2–4) 3 (1–4)	64.0 67.4 48.1	17.1 22.0 14.8	33.2 18.5 18.5	N/A N/A N/A	29.1 45.4 40.7	0–24 h	ICA, M1, M2	88.2 N/A 92.3	N/A N/A 27	7
Xue [40], 2022	Retrospective cohort study	China	EVT: 80 BMT: 449 Rescue: 43	69 (59–78) 68 (59–77) 69 (62–72)	37.5 31.6 25.6	4 (2–5) 3 (1–4) N/A	62.5 66.6 58.1	15.0 17.4 20.9	18.8 13.6 23.3	17.5 17.1 11.6	46.3 64.4 69.8	0–24 h	ICA, M1, M2, ACA	93.7 N/A 93.0 *	N/A N/A 43	7
Yedavalli [16], 2023	Retrospective cohort study	USA	EVT: 11 BMT: 35	66.5 ± 10.2 61.0 ± 13.7	27.3 54.3	3 (3–4) 2 (0–3)	81.8 80.0	27.3 31.4	36.4 11.4	27.3 22.9	54.5 14.3	0–24 h	M1, M2	N/A N/A	N/A 2	6
Cappellari [17], 2023	Retrospective cohort study	Italy	EVT: 241 BMT: 441	69.5 ± 14 68.8 ± 14.1	51.5 46.7	4 (3–5) 4 (2–5)	54.8 60.5	10.4 15.5	17.0 24.1	4.1 16.4	100.0 100.0	0–6 h	ICA, M1, M2, A1	83.2 N/A	N/A N/A	6
Palazzo [41], 2023	Retrospective cohort study	Switzer- land France	EVT: 136 BMT: 397	70 (19–96) 70 (18–96)	42.6 47.1	4 (0–5) 4 (0–5)	55.9 57.6	14.7 15.4	N/A N/A	12.5 8.3	100.0 100.0	N/A	ICA, M1, M2	95.9 76.6	N/A 29	8

* Successful recanalization rate of rescue EVT. EVT = endovascular treatment; BMT = best medical treatment; NIHSS = National Institutes of Health Stroke Scale; HTN = hypertension; DM = diabetes mellitus; AF = atrial fibrillation; tPA = tissue plasminogen activator; RoB = risk of bias; ICA = internal carotid artery; ACA = anterior cerebral artery; A1 = horizontal or pre-communicating segment of anterior cerebral artery; M1 = horizontal segment of middle cerebral artery; M2 = insular segment of middle cerebral artery; VBA = vertebrobasilar artery; BA = basilar artery; P1 = pre-communicating segment of posterior cerebral artery; N/A = not available.

## Data Availability

The datasets used and/or analyzed during the current study are available from the corresponding author on reasonable request.

## Supplementary Materials

The following supporting information can be downloaded at: https://www.mdpi.com/article/10.3390/life14101249/s1. Table S1. PRISMA Checklist. Table S2. MOOSE Checklist. Table S3. Search strategy. Table S4. Risk of bias of included studies. Table S5. GRADE approach for assessing certainty of evidence. Figure S1. Sensitivity analyses excluding low and intermediate quality study. Figure S2. Meta-analysis funnel plots and Egger’s test study.
