# Supplementary material for: Is Thrombectomy Effective for Large Vessel Occlusion Stroke Patients with Mild Symptoms? Meta-Analysis and Trial Sequential Analysis
Source: Life (Basel). 2024 Oct 1;14(10):1249. doi: 10.3390/life14101249 (PMC11508694; doi:10.3390/life14101249)
Supplement: Supplementary file 1 [file life-14-01249-s001.zip › life-3166978-supplementary.docx]

**Supplementary Information**

**Is Thrombectomy Effective for Large Vessel Occlusion Stroke Patients with Mild Symptoms? Meta‐Analysis and Trial Sequential Analysis**

Kuan-Chih Chen, Te-Wei Li, Ji-Kuan Huang, Cheng-Chieh Huang, Siang-Yan Zhang, Chih-Hung Chen, Zong-Syuan Lin, Po-Huang Chen, Hong-Jie Jhou

**Contents**

Table S1. PRISMA Checklist

Table S2. MOOSE Checklist

Table S3. Search strategy

Table S4. Risk of bias of included studies

Table S5. GRADE approach for assessing certainty of evidence

Figure S1. Sensitivity analyses excluding low and intermediate quality study

Figure S2. Meta-analysis funnel plots and Egger’s test study

**Table S1. PRISMA 2020 Checklist**

| **Topic** | **No.** | **Item** | **Location where item is reported** |
| --- | --- | --- | --- |
| **TITLE** |  |  |  |
| **Title** | 1 | Identify the report as a systematic review. | 1 |
| **ABSTRACT** |  |  |  |
| **Abstract** | 2 | See the PRISMA 2020 for Abstracts checklist | 3-4 |
| **INTRODUCTION** |  |  |  |
| **Rationale** | 3 | Describe the rationale for the review in the context of existing knowledge. | 5-6 |
| **Objectives** | 4 | Provide an explicit statement of the objective(s) or question(s) the review addresses. | 6 |
| **METHODS** |  |  |  |
| **Eligibility criteria** | 5 | Specify the inclusion and exclusion criteria for the review and how studies were grouped for the syntheses. | 7-8 |
| **Information sources** | 6 | Specify all databases, registers, websites, organisations, reference lists and other sources searched or consulted to identify studies. Specify the date when each source was last searched or consulted. | 7-8 |
| **Search strategy** | 7 | Present the full search strategies for all databases, registers and websites, including any filters and limits used. | 7-8 and Table S3 |
| **Selection process** | 8 | Specify the methods used to decide whether a study met the inclusion criteria of the review, including how many reviewers screened each record and each report retrieved, whether they worked independently, and if applicable, details of automation tools used in the process. | 7-8 |
| **Data collection process** | 9 | Specify the methods used to collect data from reports, including how many reviewers collected data from each report, whether they worked independently, any processes for obtaining or confirming data from study investigators, and if applicable, details of automation tools used in the process. | 7-8 |
| **Data items** | 10a | List and define all outcomes for which data were sought. Specify whether all results that were compatible with each outcome domain in each study were sought (e.g. for all measures, time points, analyses), and if not, the methods used to decide which results to collect. | 8-9 |
|  | 10b | List and define all other variables for which data were sought (e.g. participant and intervention characteristics, funding sources). Describe any assumptions made about any missing or unclear information. | 8-9 |
| **Study risk of bias assessment** | 11 | Specify the methods used to assess risk of bias in the included studies, including details of the tool(s) used, how many reviewers assessed each study and whether they worked independently, and if applicable, details of automation tools used in the process. | 9 |
| **Effect measures** | 12 | Specify for each outcome the effect measure(s) (e.g. risk ratio, mean difference) used in the synthesis or presentation of results. | 9 |
| **Synthesis methods** | 13a | Describe the processes used to decide which studies were eligible for each synthesis (e.g. tabulating the study intervention characteristics and comparing against the planned groups for each synthesis (item 5)). | 7-10 |
|  | 13b | Describe any methods required to prepare the data for presentation or synthesis, such as handling of missing summary statistics, or data conversions. | 7-10 |
|  | 13c | Describe any methods used to tabulate or visually display results of individual studies and syntheses. | 7-10 |
|  | 13d | Describe any methods used to synthesize results and provide a rationale for the choice(s). If meta-analysis was performed, describe the model(s), method(s) to identify the presence and extent of statistical heterogeneity, and software package(s) used. | 7-10 |
|  | 13e | Describe any methods used to explore possible causes of heterogeneity among study results (e.g. subgroup analysis, meta-regression). | 7-10 |
|  | 13f | Describe any sensitivity analyses conducted to assess robustness of the synthesized results. | 7-10, Figure S2 |
| **Reporting bias assessment** | 14 | Describe any methods used to assess risk of bias due to missing results in a synthesis (arising from reporting biases). | 9 |
| **Certainty assessment** | 15 | Describe any methods used to assess certainty (or confidence) in the body of evidence for an outcome. | 10 |
| **RESULTS** |  |  |  |
| **Study selection** | 16a | Describe the results of the search and selection process, from the number of records identified in the search to the number of studies included in the review, ideally using a flow diagram. | 11, figure S1 |
|  | 16b | Cite studies that might appear to meet the inclusion criteria, but which were excluded, and explain why they were excluded. | 11 |
| **Study characteristics** | 17 | Cite each included study and present its characteristics. | 11 and Table 1 |
| **Risk of bias in studies** | 18 | Present assessments of risk of bias for each included study. | Table S4 |
| **Results of individual studies** | 19 | For all outcomes, present, for each study: (a) summary statistics for each group (where appropriate) and (b) an effect estimate and its precision (e.g. confidence/credible interval), ideally using structured tables or plots. | 11-15 |
| **Results of syntheses** | 20a | For each synthesis, briefly summarise the characteristics and risk of bias among contributing studies. | 14-15 |
|  | 20b | Present results of all statistical syntheses conducted. If meta-analysis was done, present for each the summary estimate and its precision (e.g. confidence/credible interval) and measures of statistical heterogeneity. If comparing groups, describe the direction of the effect. | 11-15 |
|  | 20c | Present results of all investigations of possible causes of heterogeneity among study results. | 14-15 |
|  | 20d | Present results of all sensitivity analyses conducted to assess the robustness of the synthesized results. | 14-15, Figure S2 |
| **Reporting biases** | 21 | Present assessments of risk of bias due to missing results (arising from reporting biases) for each synthesis assessed. | Table S4 |
| **Certainty of evidence** | 22 | Present assessments of certainty (or confidence) in the body of evidence for each outcome assessed. | 14-15, Table S5 |
| **DISCUSSION** |  |  |  |
| **Discussion** | 23a | Provide a general interpretation of the results in the context of other evidence. | 16 |
|  | 23b | Discuss any limitations of the evidence included in the review. | 18 |
|  | 23c | Discuss any limitations of the review processes used. | 18 |
|  | 23d | Discuss implications of the results for practice, policy, and future research. | 18-19 |
| **OTHER INFORMATION** |  |  |  |
| **Registration and protocol** | 24a | Provide registration information for the review, including register name and registration number, or state that the review was not registered. | 7 |
|  | 24b | Indicate where the review protocol can be accessed, or state that a protocol was not prepared. | 7 |
|  | 24c | Describe and explain any amendments to information provided at registration or in the protocol. | 7 |
| **Support** | 25 | Describe sources of financial or non-financial support for the review, and the role of the funders or sponsors in the review. | 20 |
| **Competing interests** | 26 | Declare any competing interests of review authors. | 20 |
| **Availability of data, code and other materials** | 27 | Report which of the following are publicly available and where they can be found: template data collection forms; data extracted from included studies; data used for all analyses; analytic code; any other materials used in the review. | 7 |

*From:* Page MJ, McKenzie JE, Bossuyt PM, Boutron I, Hoffmann TC, Mulrow CD, et al. The PRISMA 2020 statement: an updated guideline for reporting systematic reviews. MetaArXiv. 2020, September 14. DOI: 10.31222/osf.io/v7gm2. For more information, visit: [www.prisma-statement.org](file:///C:\Users\user\Downloads\www.prisma-statement.org)

**Table S2. MOOSE Checklist**

| **Item No** | **Recommendation** | **Reported on Page No** |
| --- | --- | --- |
| Reporting of background should include | | |
| 1 | Problem definition | 5-6 |
| 2 | Hypothesis statement | 6 |
| 3 | Description of study outcome(s) | 6 |
| 4 | Type of exposure or intervention used | 5-6 |
| 5 | Type of study designs used | 5-6 |
| 6 | Study population | 5-6 |
| Reporting of search strategy should include | | |
| 7 | Qualifications of searchers (eg, librarians and investigators) | 7-8 |
| 8 | Search strategy, including time period included in the synthesis and keywords | 7 |
| 9 | Effort to include all available studies, including contact with authors | 7 |
| 10 | Databases and registries searched | 7-8 |
| 11 | Search software used, name and version, including special features used (eg, explosion) | Manual |
| 12 | Use of hand searching (eg, reference lists of obtained articles) | 7-8 |
| 13 | List of citations located and those excluded, including justification | 7-8 |
| 14 | Method of addressing articles published in languages other than English | NA |
| 15 | Method of handling abstracts and unpublished studies | 7-8 |
| 16 | Description of any contact with authors | 7 |
| Reporting of methods should include | | |
| 17 | Description of relevance or appropriateness of studies assembled for assessing the hypothesis to be tested | 7-8 |
| 18 | Rationale for the selection and coding of data (eg, sound clinical principles or convenience) | 7-8 |
| 19 | Documentation of how data were classified and coded (eg, multiple raters, blinding and interrater reliability) | 7-9 |
| 20 | Assessment of confounding (eg, comparability of cases and controls in studies where appropriate) | Table S5 |
| 21 | Assessment of study quality, including blinding of quality assessors, stratification or regression on possible predictors of study results | Table S5 |
| 22 | Assessment of heterogeneity | 9 |
| 23 | Description of statistical methods (eg, complete description of fixed or random effects models, justification of whether the chosen models account for predictors of study results, dose-response models, or cumulative meta-analysis) in sufficient detail to be replicated | 9 |
| 24 | Provision of appropriate tables and graphics | 21-22 |

**Table S3. Search Strategy**

| Database | Search detail |
| --- | --- |
| PubMed | **#1** (((("Brain Infarction"[MeSH Terms] OR "Ischemic Stroke"[MeSH Terms] OR "Cerebrovascular Disorders"[Mesh]) OR ("Brain Infarction" OR "Ischemic Stroke" OR "Cerebrovascular Disorders" or "large vessel occlusion")) OR ((Brain Infarction) OR (Ischemic Stroke) OR (Cerebrovascular Disorders) or (large vessel occlusion))) OR ((Brain Infarction*) OR (Ischemic Stroke*) OR (Cerebrovascular Disorders*) or (large vessel occlusion*))) OR (("Brain Infarction*") OR ("Ischemic Stroke*") OR ("Cerebrovascular Disorders*") or ("large vessel occlusion*"))  **#2** "Mild" or (Mild) or (Mild*) or ("Mild") or "Minor" or (Minor) or (Minor*) or ("Minor") or "Low NIHSS" or (Low NIHSS) or (Low NIHSS*) or ("Low NIHSS") or "NIHSS≤5" or (NIHSS≤5) or (NIHSS≤5*) or ("NIHSS≤5") or "NIHSS<6" or (NIHSS<6) or (NIHSS<6*) or ("NIHSS<6") or (Low National Institute Health Stroke Scale) or (Low National Institute Health Stroke Scale*) or (National Institute Health Stroke Scale≤5) or (National Institute Health Stroke Scale≤5*) or (National Institute Health Stroke Scale<6) or (National Institute Health Stroke Scale<6*)  **#3** (((("Endovascular Procedures"[MeSH Terms] OR "Thrombectomy"[MeSH Terms]) OR ("Endovascular Procedures" OR "Thrombectomy")) OR ((Endovascular Procedures) OR (Thrombectomy))) OR ((Endovascular Procedures*) OR (Thrombectomy*))) OR (("Endovascular Procedures*") OR ("Thrombectomy*"))  **#4** #1 AND #2 AND #3 |
| Cochrane | **#1** (Brain Infarction):ti,ab,kw  **#2** (Ischemic Stroke):ti,ab,kw  **#3** MeSH descriptor: [Brain Infarction] explode all trees  **#4** MeSH descriptor: [Ischemic Stroke] explode all trees  **#5** ("Brain Infarction"):ti,ab,kw  **#6** ("Ischemic Stroke"):ti,ab,kw  **#7** ((Brain Infarction*)):ti,ab,kw  **#8** ((Ischemic Stroke*)):ti,ab,kw  **#9** #1 or #2 or #3 or #4 or #5 or #6 or #7 or #8  **#10** (mild):ti,ab,kw  **#11** (minor):ti,ab,kw  **#12** (low NIHSS):ti,ab,kw  **#13** (nihss≤5):ti,ab,kw  **#14** #10 or #11 or #12 or #13  **#15** #9 and #14  **#16** MeSH descriptor: [Endovascular Procedures] explode all trees  **#17** MeSH descriptor: [Thrombectomy] explode all trees  **#18** ("Endovascular Procedures"):ti,ab,kw  **#19** ("Thrombectomy"):ti,ab,kw  **#20** ((Endovascular Procedures)):ti,ab,kw  **#21** ((Thrombectomy)):ti,ab,kw  **#22** ((Endovascular Procedures*)):ti,ab,kw  **#23** ((Thrombectomy*)):ti,ab,kw  **#24** #18 or #19 or #20 or #21 or #22 or #23  **#25** #15 and #24 |
| EmBase | **#1** 'ischemic stroke'/exp OR 'ischaemic stroke' OR  'ischemic stroke' OR 'brain infarction'/exp OR 'brain cortex infarct' OR 'brain cortex infarction' OR 'brain infarct' OR 'brain infarction' OR 'cerebral cortex infarct' OR 'cerebral cortex infarction' OR 'cerebral infarct' OR 'cerebral infarction' OR 'cerebrovascular infarct' OR 'cerebrovascular infarction' OR 'cortical infarct' OR 'cortical infarction' OR 'hemisphere infarct' OR 'hemisphere infarction' OR 'hemispheric infarct' OR 'hemispheric infarction' OR 'infarction, brain' OR 'silent brain infarction'  **#2** 'thrombectomy'/exp OR thrombectomy OR  'thrombectomy'  **#3** mild OR 'minor'/exp OR minor OR 'low nihss' **#4** #1 AND #2 AND #3 |

**Table S4. Risk of bias of included studies**

| Newcastle-Ottawa Scale quality assessment scale for cohort studies | | | | | | | | |
| --- | --- | --- | --- | --- | --- | --- | --- | --- |
| First author, year | Representativeness of the exposed cohort | Selection of  the nonexposed cohort | Ascertainment of exposure | Demonstration that outcome of interest was not present at start of study | Comparability of cohorts on  the basis of  the design  or analysis | Assessment of outcome | Was follow-up long enough for outcomes to occur | Adequacy of follow up of cohorts |
| Ros, 2019 | **🟑** | **🟑** | **🟑** | **🟑** | **🟑🟑** | **🟑** | **🟑** | **🟑** |
| Goyal, 2020 | **🟑** | **🟑** | **🟑** | **🟑** | **🟑** | **🟑** | **🟑** | **🟑** |
| Abbas, 2022 | **🟑** | **🟑** | **🟑** |  | **🟑🟑** | **🟑** | **🟑** |  |
| Kim, 2022 | **🟑** | **🟑** | **🟑** |  | **🟑🟑** | **🟑** | **🟑** |  |
| Sarraj, 2022 | **🟑** | **🟑** | **🟑** |  | **🟑** | **🟑** | **🟑** | **🟑** |
| Xue, 2022 | **🟑** | **🟑** | **🟑** | **🟑** | **🟑** | **🟑** | **🟑** |  |
| Yedavalli, 2023 | **🟑** | **🟑** | **🟑** |  | **🟑** | **🟑** | **🟑** |  |
| Wang, 2020 | **🟑** | **🟑** | **🟑** | **🟑** | **🟑🟑** | **🟑** | **🟑** |  |
| Seners, 2021 | **🟑** | **🟑** | **🟑** |  | **🟑** | **🟑** | **🟑** | **🟑** |
| Cappellari, 2023 | **🟑** | **🟑** | **🟑** |  | **🟑** | **🟑** | **🟑** |  |
| Liu, 2021 | **🟑** | **🟑** | **🟑** | **🟑** | **🟑** | **🟑** | **🟑** | **🟑** |
| Nagel, 2018 | **🟑** | **🟑** | **🟑** |  | **🟑** | **🟑** | **🟑** |  |
| Urra, 2015 | **🟑** | **🟑** | **🟑** | **🟑** | **🟑🟑** | **🟑** | **🟑** |  |
| Palazzo, 2023 | **🟑** | **🟑** | **🟑** | **🟑** | **🟑** | **🟑** | **🟑** | **🟑** |
| Overall score: 0-3 points = low quality; 4-6 points = intermediate quality; 7-9 points = high quality. | | | | | | | | |
|  |  |  |  |  |  |  |  |  |

**Table S5. GRADE approach for assessing certainty of evidence**

**Crossover group**

| **Certainty assessment** | | | | | | | **№ of patients** | | **Effect** | | **Certainty** | **Importance** |
| --- | --- | --- | --- | --- | --- | --- | --- | --- | --- | --- | --- | --- |
| **№ of studies** | **Study design** | **Risk of bias** | **Inconsistency** | **Indirectness** | **Imprecision** | **Other considerations** | **[intervention]** | **[comparison]** | **Relative (95% CI)** | **Absolute (95% CI)** |  |  |
| **Excellent functional outcome at 3 months** | | | | | | | | | | | | |
| 7 | non-randomised studies | not serious | serious^a^ | not serious | serious^b^ | none | 410/706 (58.1%) | 972/1727 (56.3%) | **OR 1.02** (0.72 to 1.45) | **5 more per 1,000** (from 82 fewer to 88 more) | ⨁◯◯◯ Very low | CRITICAL |
| **Good functional outcome at 3 months** | | | | | | | | | | | | |
| 8 | non-randomised studies | not serious | not serious | not serious | serious^b^ | none | 538/747 (72.0%) | 1270/1769 (71.8%) | **OR 1.04** (0.73 to 1.47) | **8 more per 1,000** (from 68 fewer to 71 more) | ⨁◯◯◯ Very low | CRITICAL |
| **Favorable functional outcome at 3 months** | | | | | | | | | | | | |
| 2 | non-randomised studies | not serious | very serious^c^ | not serious | serious^b^ | none | 293/347 (84.4%) | 225/271 (83.0%) | **OR 0.71** (0.16 to 3.15) | **54 fewer per 1,000** (from 391 fewer to 109 more) | ⨁◯◯◯ Very low | CRITICAL |
| **Symptomatic intracrainal hemorrhage** | | | | | | | | | | | | |
| 7 | non-randomised studies | not serious | not serious | not serious | not serious | very strong association | 57/598 (9.5%) | 12/835 (1.4%) | **OR 5.18** (2.54 to 10.56) | **56 more per 1,000** (from 21 more to 119 more) | ⨁⨁⨁⨁ High | CRITICAL |
| **Mortality at 3 months** | | | | | | | | | | | | |
| 7 | non-randomised studies | not serious | not serious | not serious | serious^b^ | none | 36/715 (5.0%) | 68/1744 (3.9%) | **OR 1.47** (0.93 to 2.33) | **17 more per 1,000** (from 3 fewer to 47 more) | ⨁◯◯◯ Very low | CRITICAL |

**CI:** confidence interval; **OR:** odds ratio

**Explanations**

a. There was substantial heterogeneity, and some variability in the point estimates in the forest plots.

b. The results of the trial sequential analysis indicated inconclusive evidence, as the cumulative z-statistic line did not cross either the conventional boundary or the trial sequential monitoring boundary.

c. There was considerable heterogeneity, and some variability in the point estimates in the forest plots.

**Non-crossover group**

| **Certainty assessment** | | | | | | | **№ of patients** | | **Effect** | | **Certainty** | **Importance** |
| --- | --- | --- | --- | --- | --- | --- | --- | --- | --- | --- | --- | --- |
| **№ of studies** | **Study design** | **Risk of bias** | **Inconsistency** | **Indirectness** | **Imprecision** | **Other considerations** | **[intervention]** | **[comparison]** | **Relative (95% CI)** | **Absolute (95% CI)** |  |  |
| **Excellent functional outcome at 3 months** | | | | | | | | | | | | |
| 9 | non-randomised studies | not serious | very serious^a^ | not serious | not serious | none | 679/1044 (65.0%) | 1098/2045 (53.7%) | **OR 1.62** (1.13 to 2.32) | **116 more per 1,000** (from 30 more to 192 more) | ⨁◯◯◯ Very low | CRITICAL |
| **Good functional outcome at 3 months** | | | | | | | | | | | | |
| 9 | non-randomised studies | not serious | very serious^a^ | not serious | not serious | none | 809/1044 (77.5%) | 1375/2045 (67.2%) | **OR 1.66** (1.06 to 2.60) | **101 more per 1,000** (from 13 more to 170 more) | ⨁◯◯◯ Very low | CRITICAL |
| **Favorable functional outcome at 3 months** | | | | | | | | | | | | |
| 3 | non-randomised studies | not serious | very serious^a^ | not serious | serious^b^ | none | 562/663 (84.8%) | 780/1092 (71.4%) | **OR 1.91** (0.84 to 4.31) | **113 more per 1,000** (from 37 fewer to 201 more) | ⨁◯◯◯ Very low | CRITICAL |
| **Symptomatic intracrainal hemorrhage** | | | | | | | | | | | | |
| 7 | non-randomised studies | not serious | not serious | not serious | not serious | strong association | 60/897 (6.7%) | 34/1613 (2.1%) | **OR 3.02** (1.35 to 6.75) | **40 more per 1,000** (from 7 more to 106 more) | ⨁⨁⨁◯ Moderate | CRITICAL |
| **Mortality at 3 months** | | | | | | | | | | | | |
| 8 | non-randomised studies | not serious | not serious | not serious | not serious | none | 52/1016 (5.1%) | 105/2016 (5.2%) | **OR 1.09** (0.66 to 1.80) | **4 more per 1,000** (from 17 fewer to 38 more) | ⨁⨁◯◯ Low | CRITICAL |

**CI:** confidence interval; **OR:** odds ratio

**Explanations**

a. There was considerable heterogeneity, and some variability in the point estimates in the forest plots.

b. The results of the trial sequential analysis indicated inconclusive evidence, as the cumulative z-statistic line did not cross either the conventional boundary or the trial sequential monitoring boundary.

**Figure S1. Sensitivity analyses excluding low and intermediate quality study**


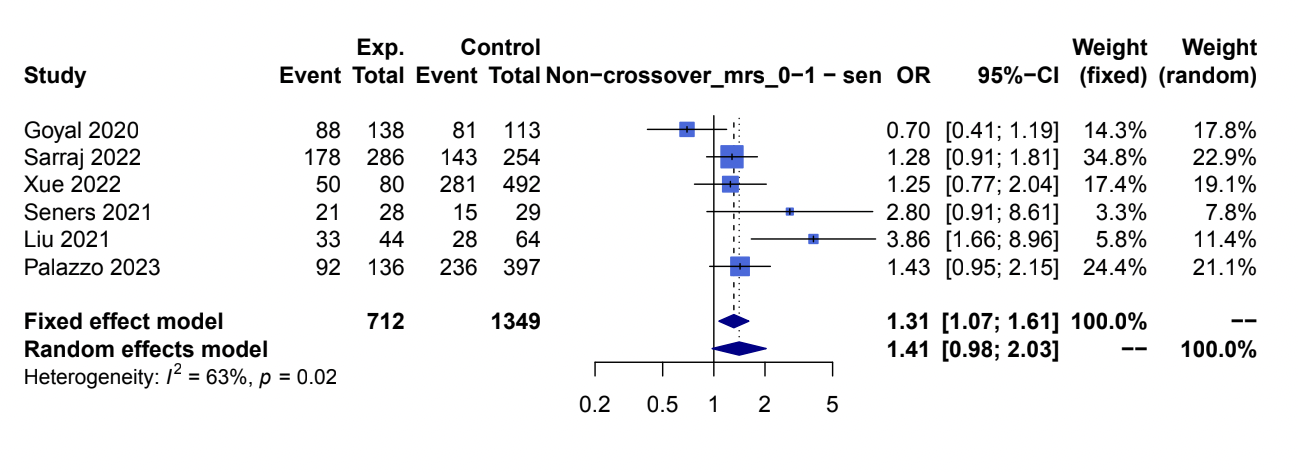

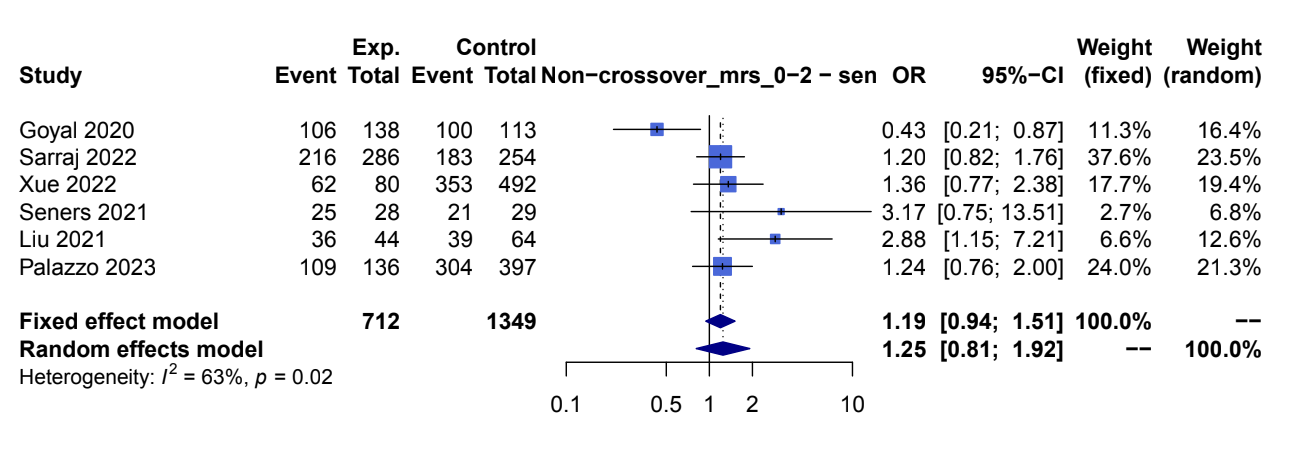

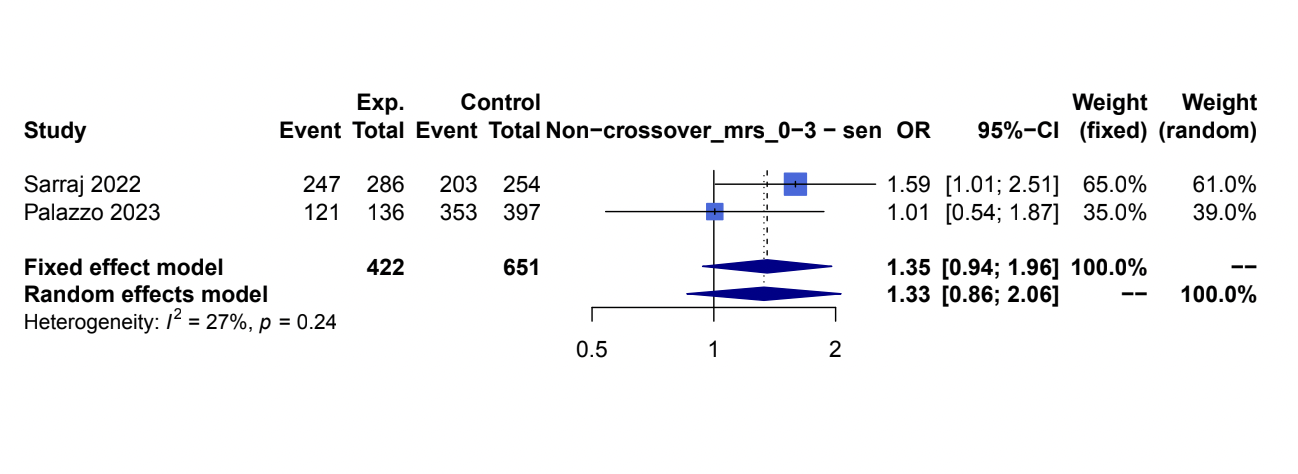

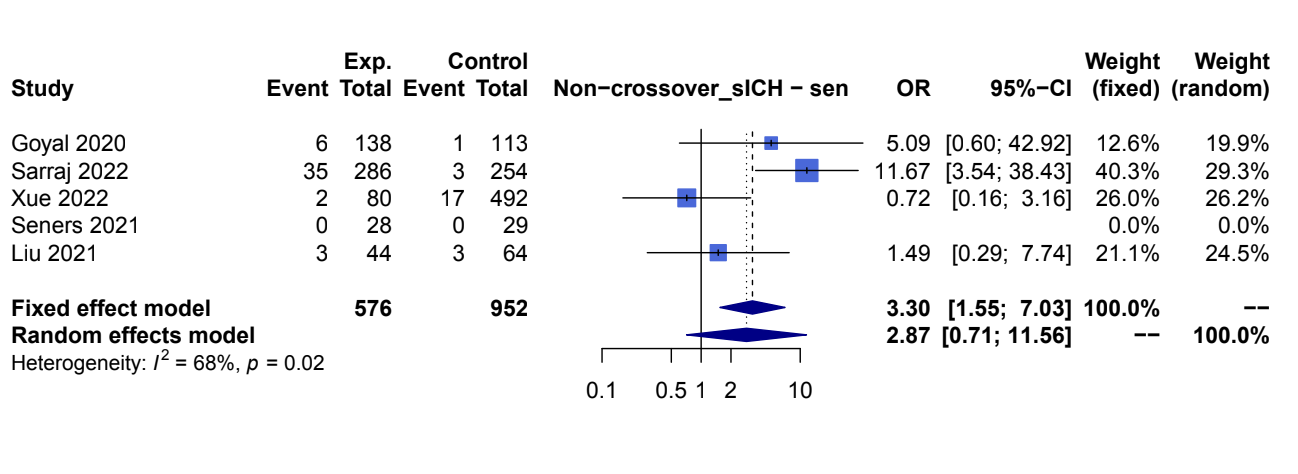

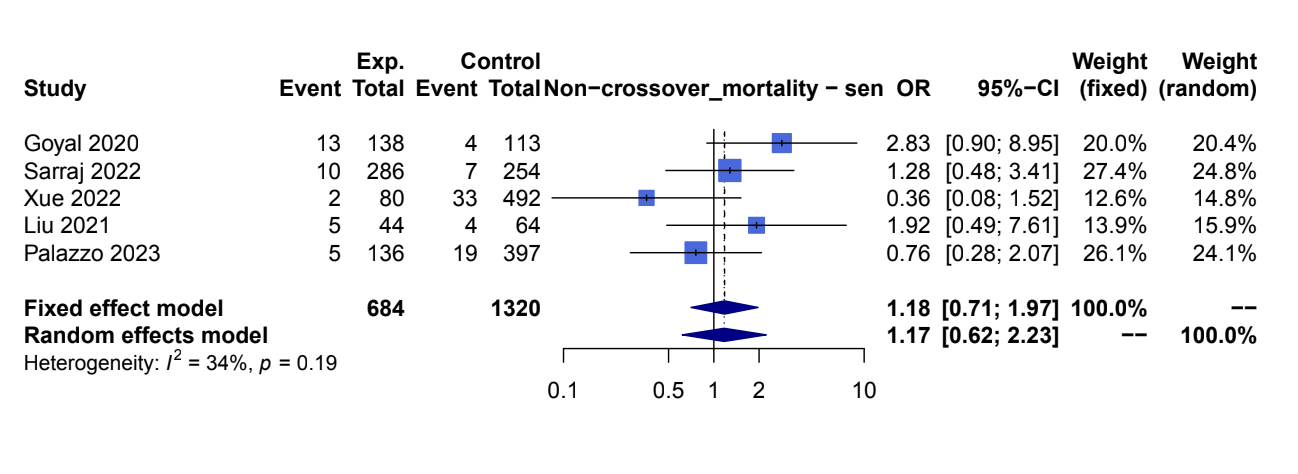


**D**

**C**

**B**

**A**

(A) Excellent functional outcomes at 3 months (B) Good functional outcome at 3 months(C) Favorable outcome at 3 months (D) Symptomatic intracerebral hemorrhage (E) Mortality at 3 months

**E**

**Figure S2. Meta-analysis funnel plots and Egger’s test**

**Crossover group**


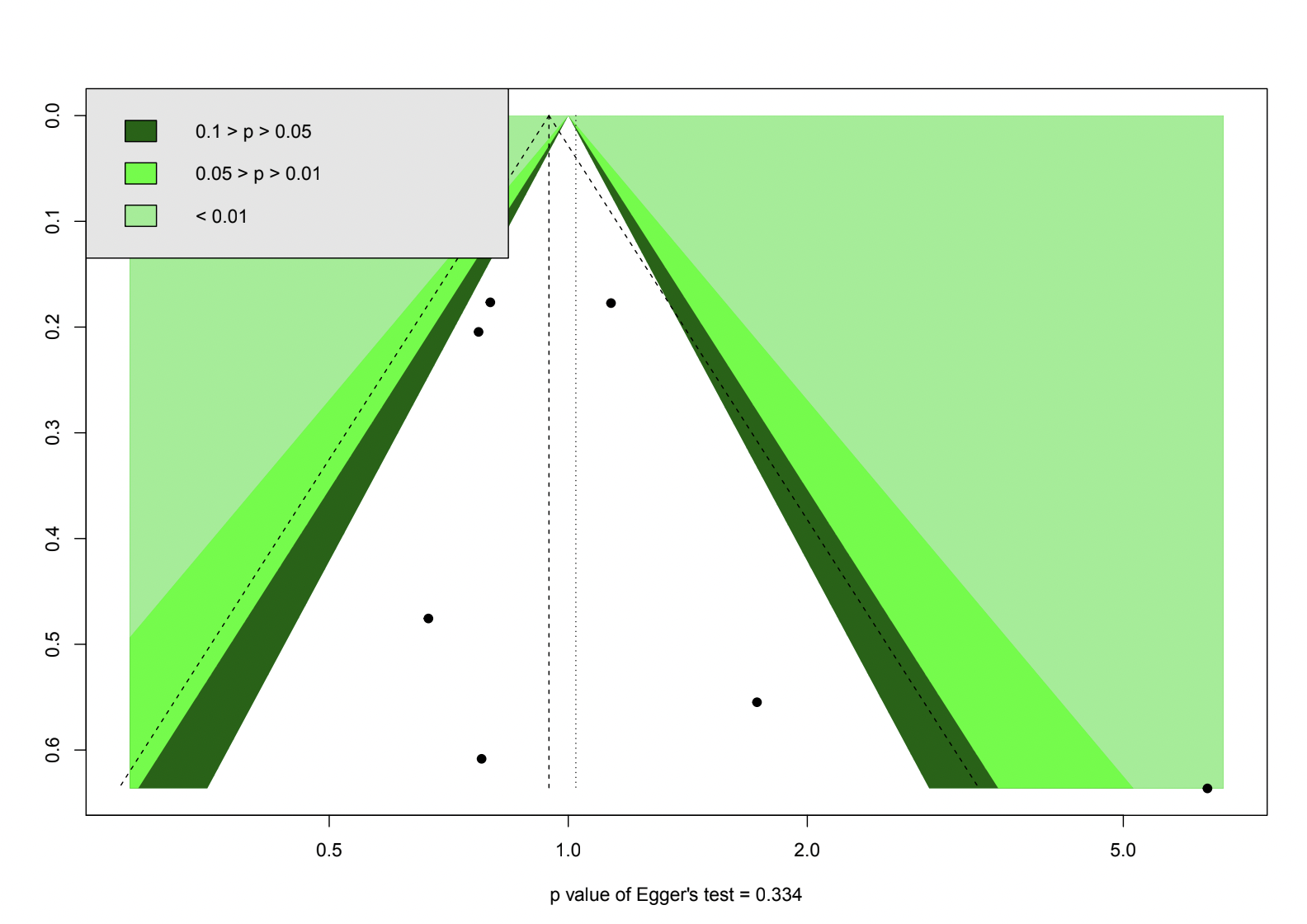


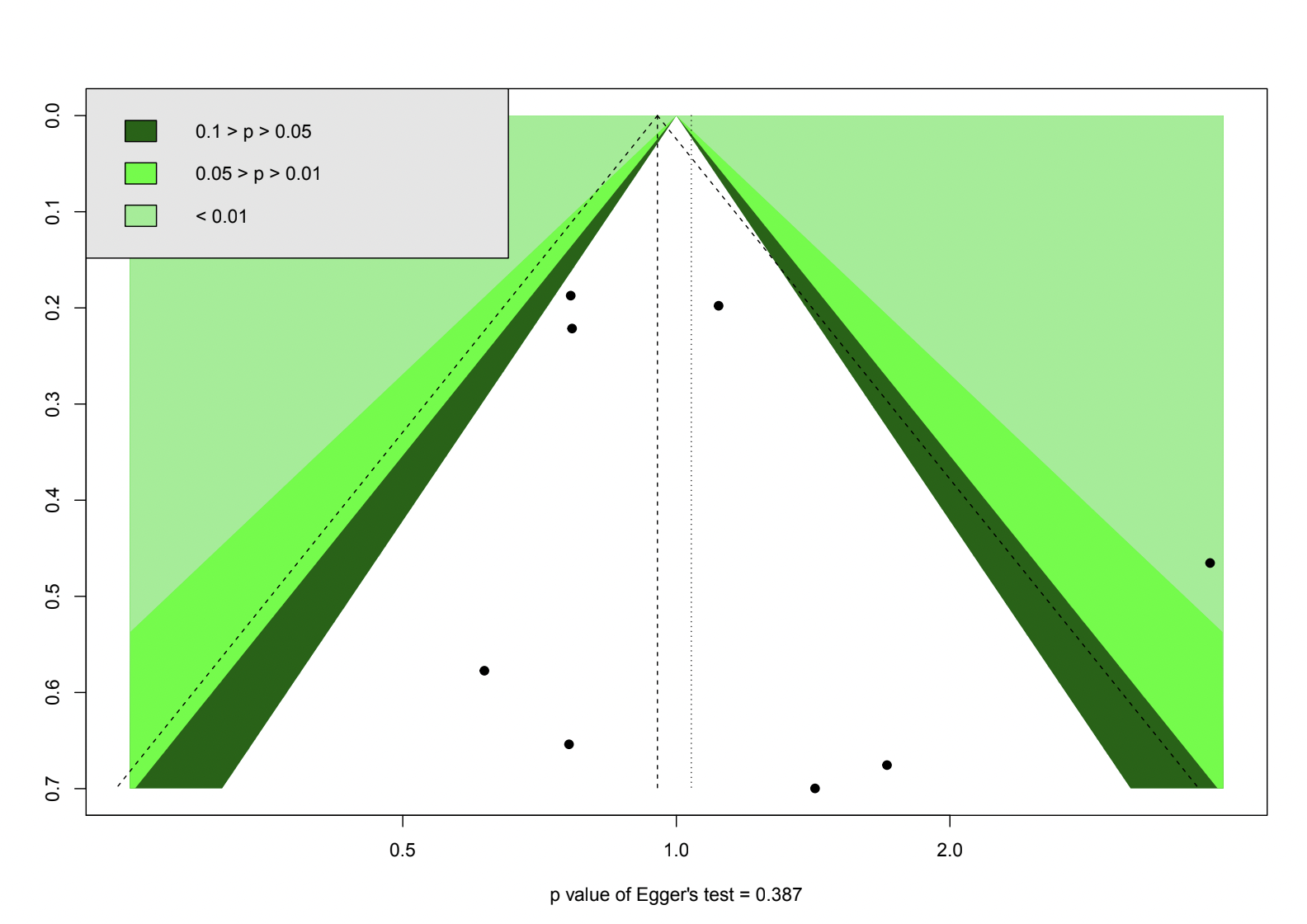


Figure S2 Funnel plot in outcome for good functional outcomes at 3 months in crossover group


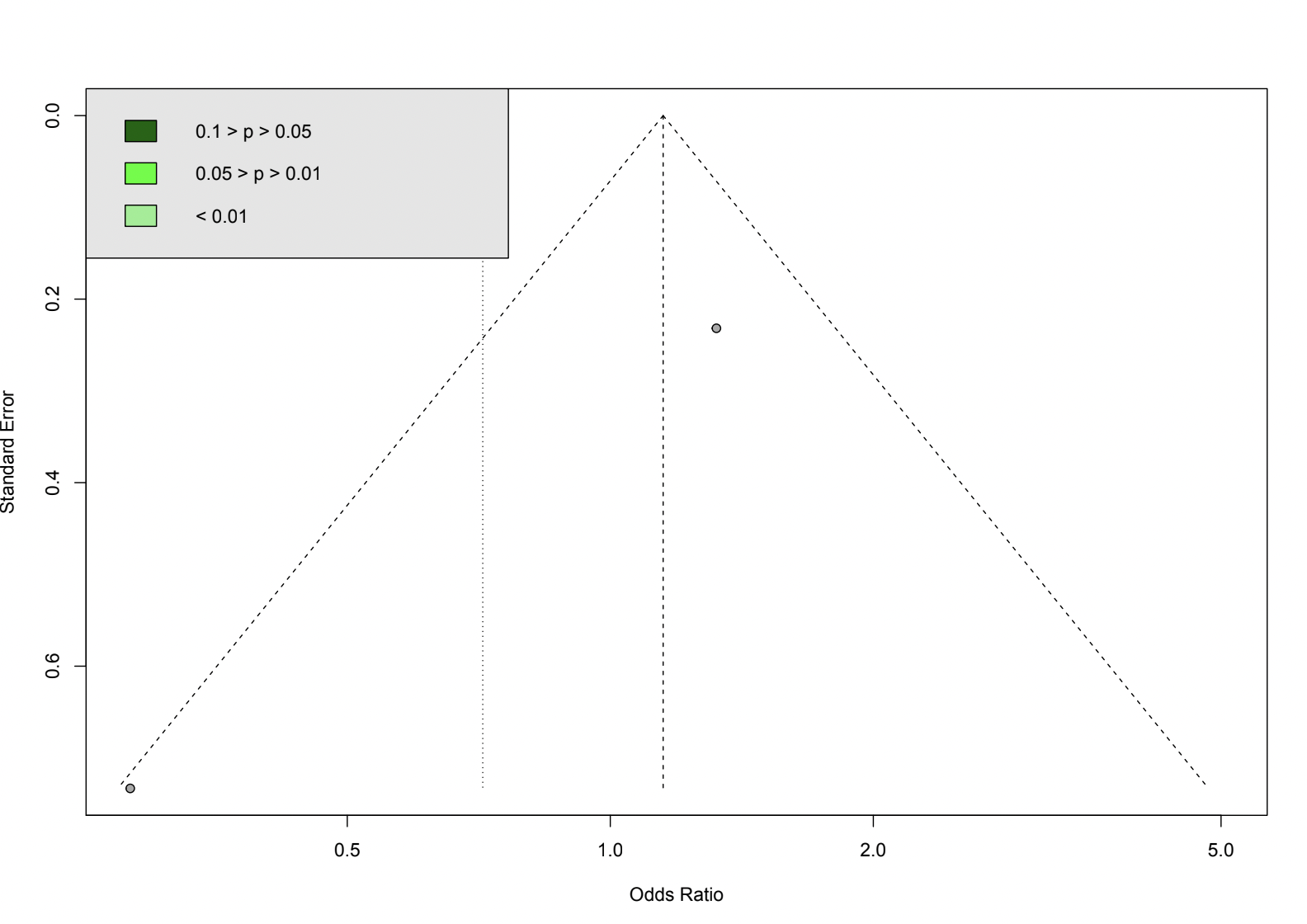


Figure S2 Funnel plot in outcome for favorable functional outcomes at 3 months in crossover group


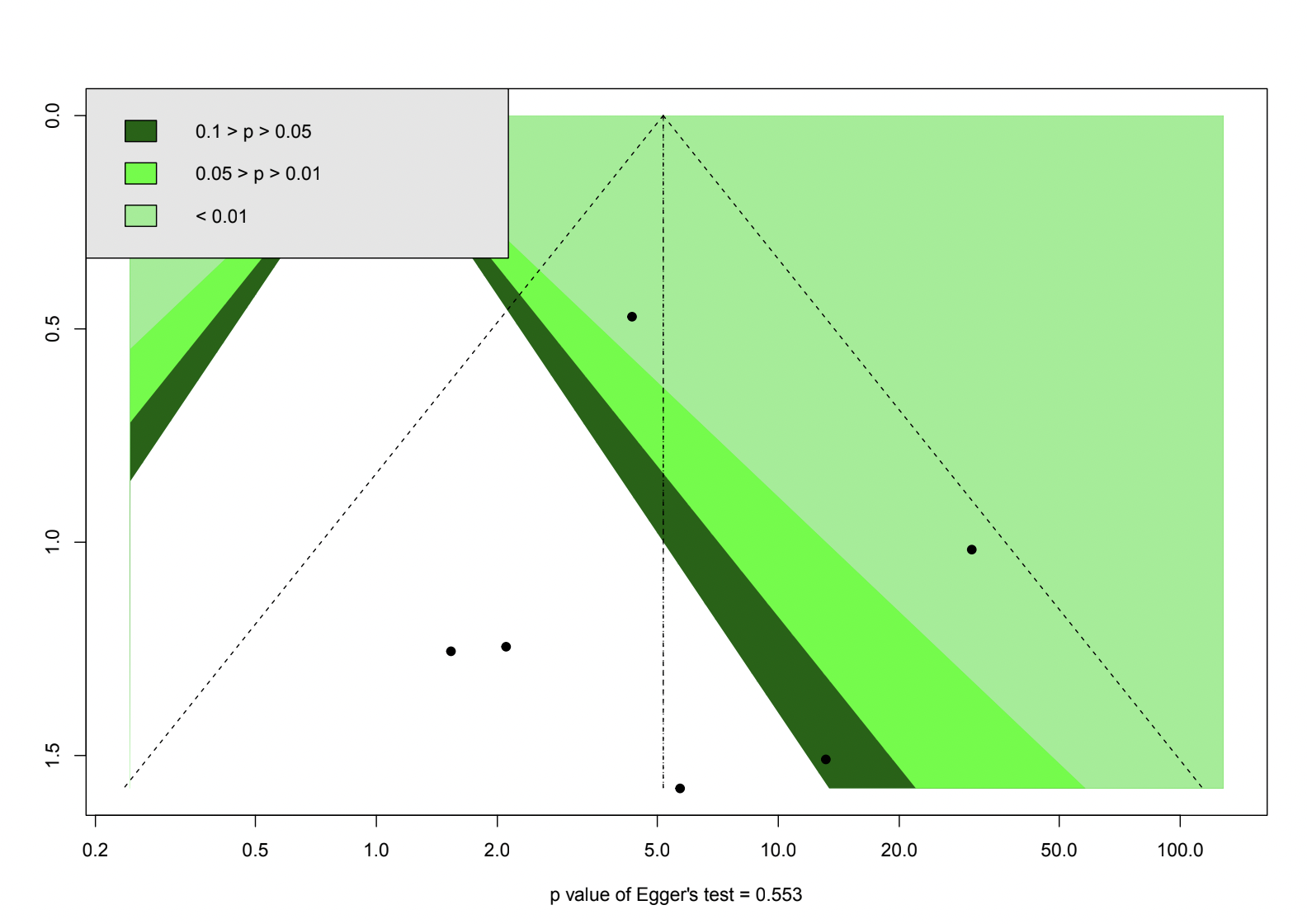


Figure S2 Funnel plot in outcome for symptomatic intracerebral hemorrhage in crossover group


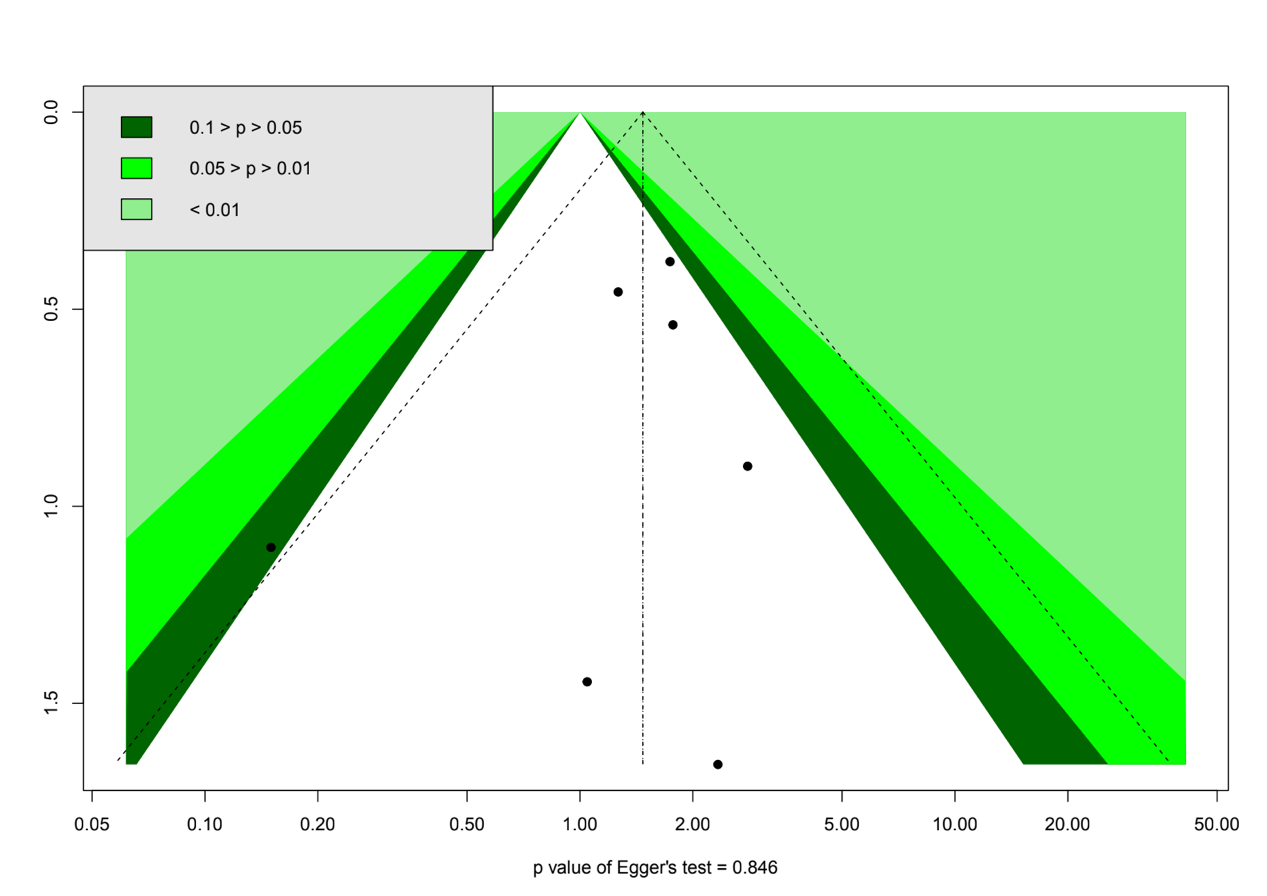


Figure S2 Funnel plot in outcome for mortality at 3 months in crossover group


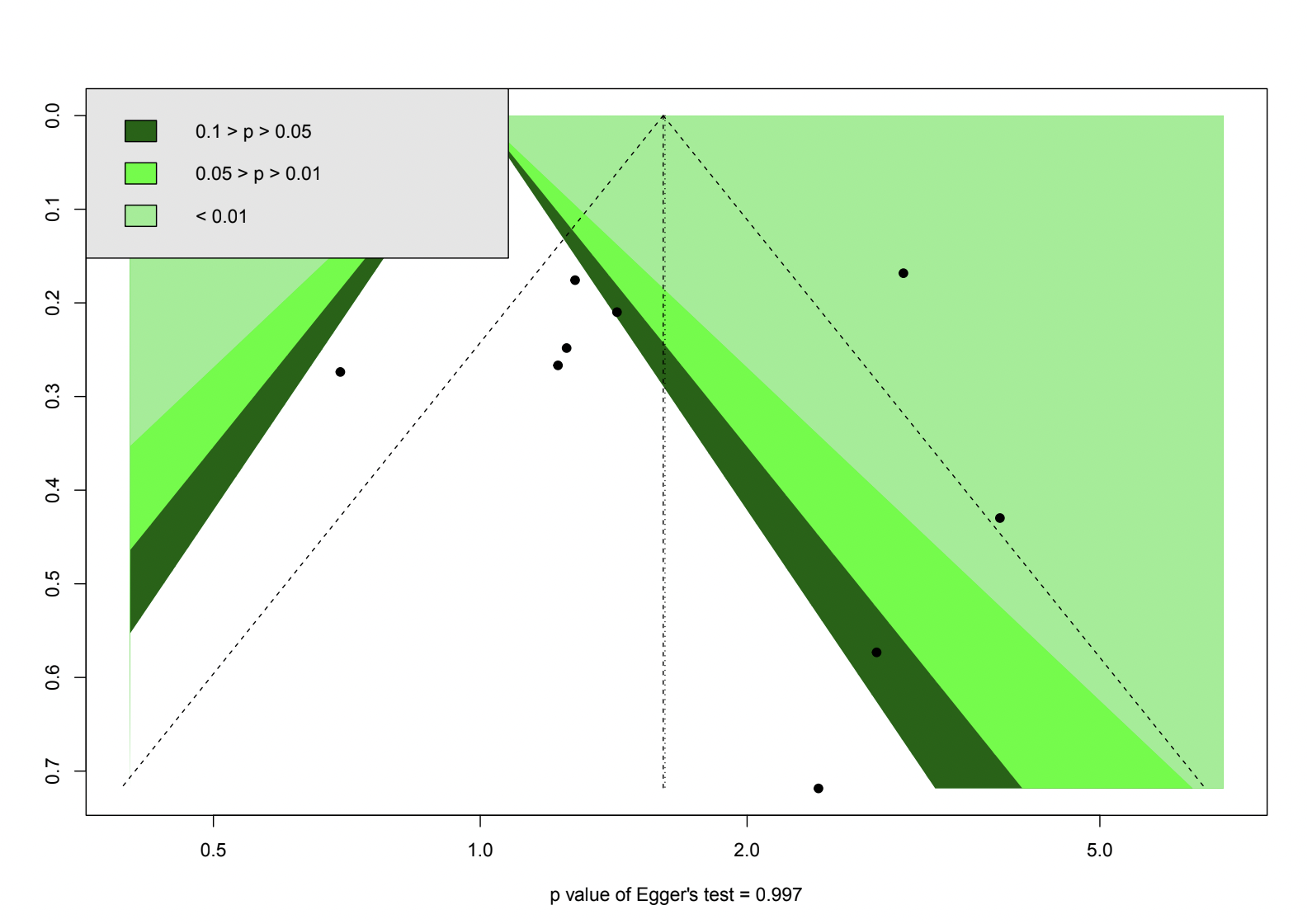
**Non-crossover group**

Figure S2 Funnel plot in outcome for excellent functional outcomes at 3 months in non-crossover group


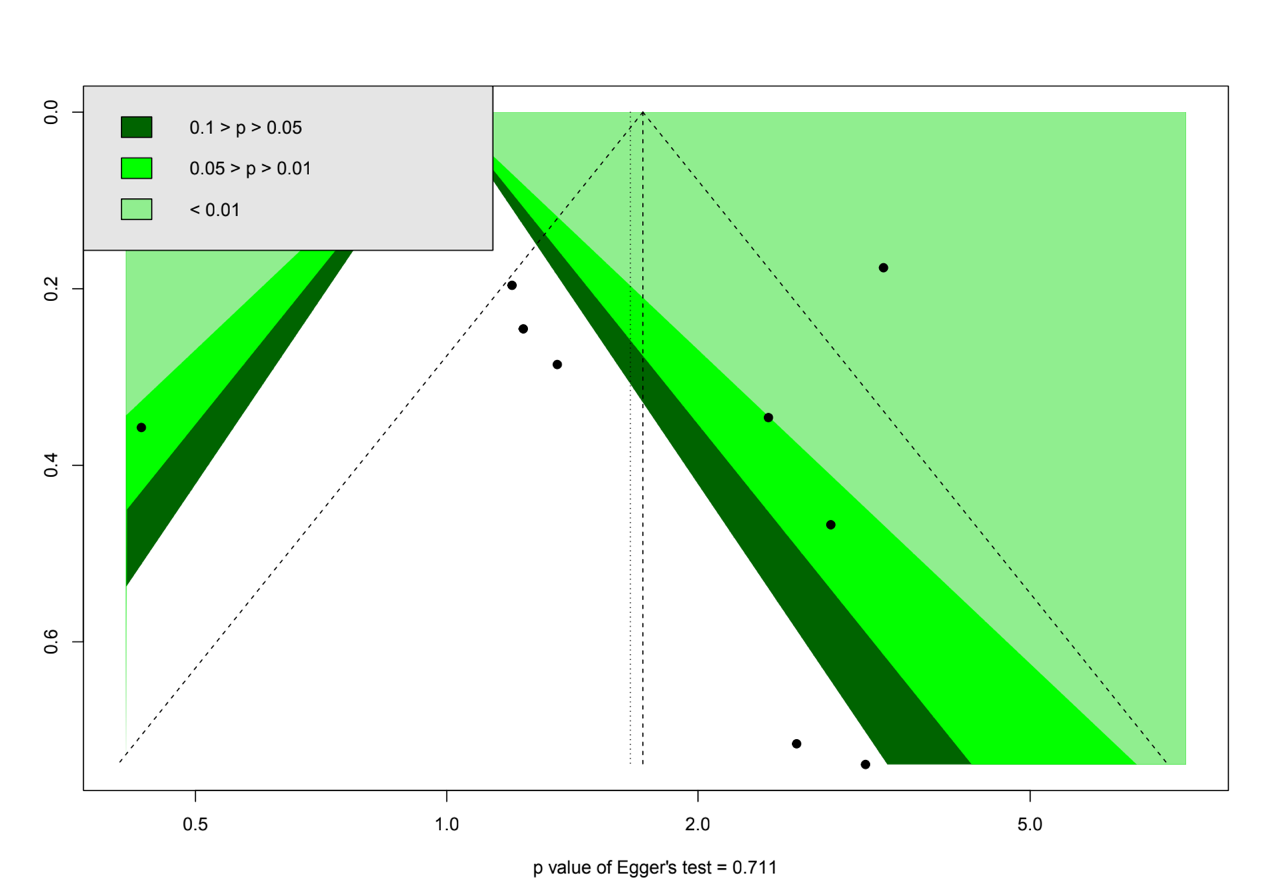


Figure S2 Funnel plot in outcome for good functional outcomes at 3 months in non-crossover group


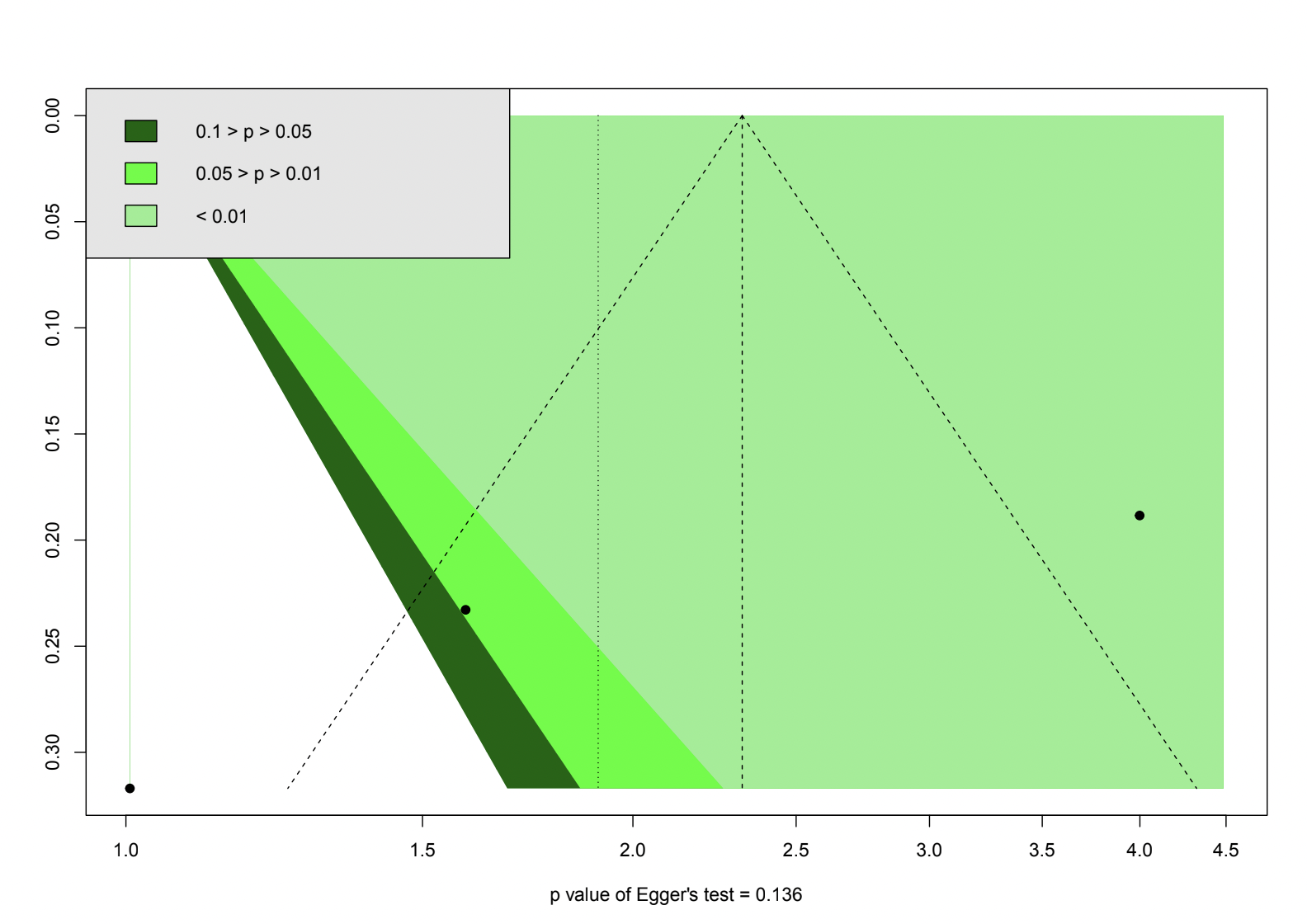


Figure S2 Funnel plot in outcome for favorable functional outcomes at 3 months in non-crossover group


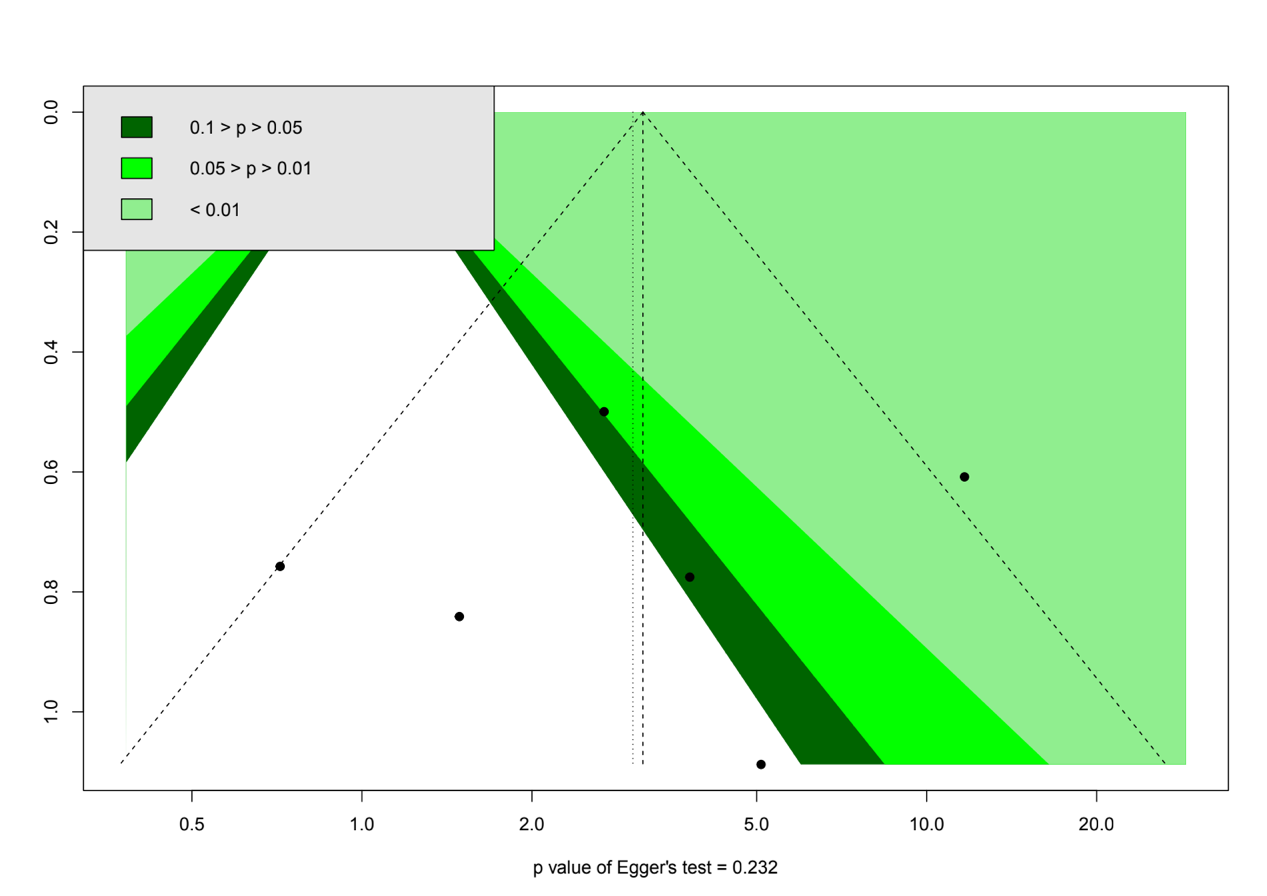


Figure S2 Funnel plot in outcome for symptomatic intracerebral hemorrhage in non-crossover group


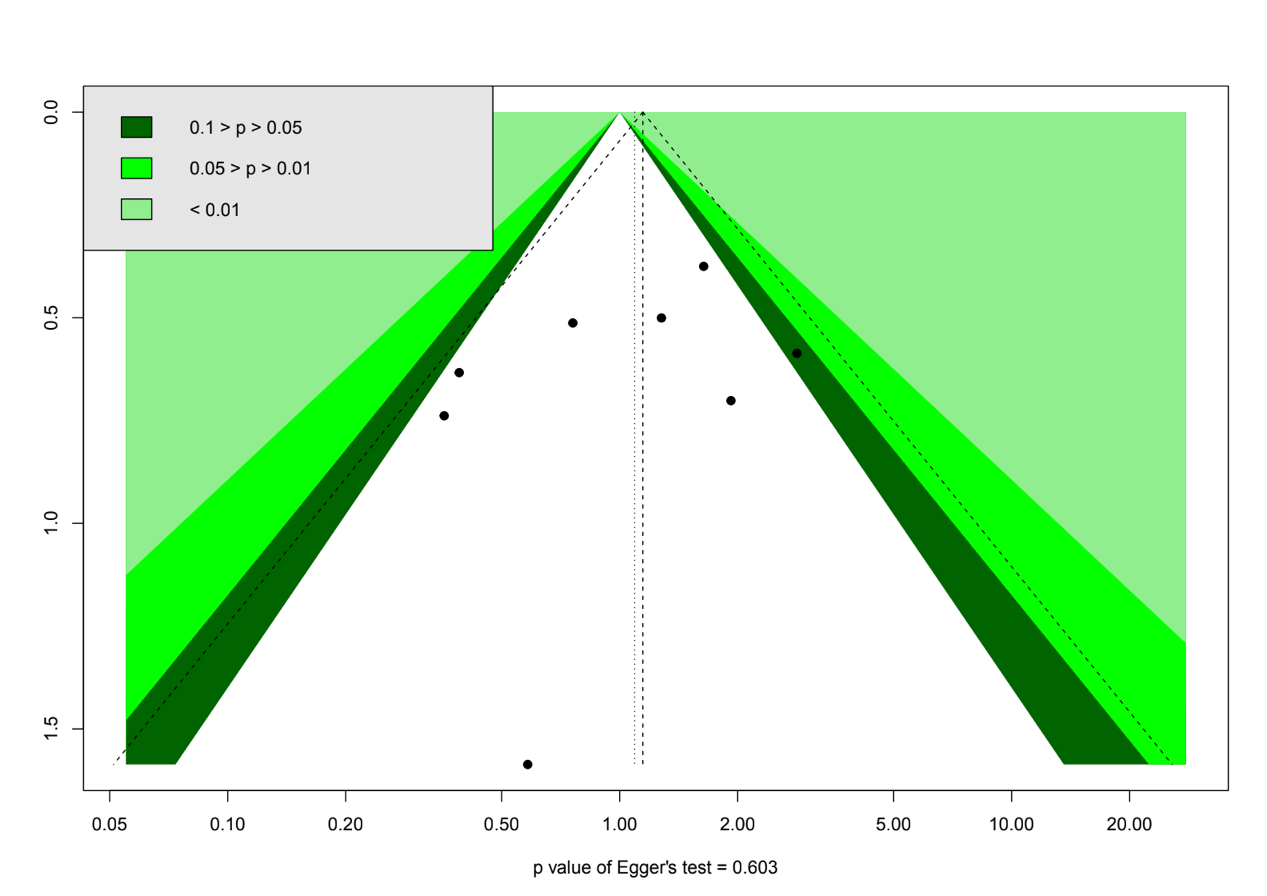


Figure S2 Funnel plot in outcome for mortality at 3 months in non-crossover group
